# A universal GlycoDesign for lysosomal replacement enzymes to improve circulation time and biodistribution

**DOI:** 10.3389/fbioe.2023.1128371

**Published:** 2023-02-24

**Authors:** Yen-Hsi Chen, Weihua Tian, Makiko Yasuda, Zilu Ye, Ming Song, Ulla Mandel, Claus Kristensen, Lorenzo Povolo, André R. A. Marques, Tomislav Čaval, Albert J. R. Heck, Julio Lopes Sampaio, Ludger Johannes, Takahiro Tsukimura, Robert Desnick, Sergey Y. Vakhrushev, Zhang Yang, Henrik Clausen

**Affiliations:** ^1^ Copenhagen Center for Glycomics, Department of Cellular and Molecular Medicine, Faculty of Health and Medical Sciences, University of Copenhagen, Copenhagen, Denmark; ^2^ GlycoDisplay ApS, Copenhagen, Denmark; ^3^ Department of Biotechnology and Biomedicine, Technical University of Denmark, Lyngby, Denmark; ^4^ Department of Genetics and Genomic Sciences, Icahn School of Medicine at Mount Sinai, New York, NY, United States; ^5^ Novo Nordisk Foundation Center for Protein Research, Proteomics Program, Faculty of Health and Medical Sciences, University of Copenhagen, Copenhagen, Denmark; ^6^ Biochemisches Institut, CAU Kiel, Kiel, Germany; ^7^ Biomolecular Mass Spectrometry and Proteomics, Bijvoet Center for Biomolecular Research and Utrecht Institute for Pharmaceutical Sciences, Science4Life, Utrecht University and Netherlands Proteomics Centre, Utrecht, Netherlands; ^8^ Institut Curie, PSL Research University, Cellular and Chemical Biology, U1143 INSERM, UMR3666 CNRS, Paris, France; ^9^ Department of Functional Bioanalysis, Meiji Pharmaceutical University, Tokyo, Japan; ^10^ Novo Nordisk AS, Copenhagen, Denmark

**Keywords:** glycoengineering, enzyme replacement therapy, lysosomal storage disease, glycoprotein therapeutics, bioengineering

## Abstract

Currently available enzyme replacement therapies for lysosomal storage diseases are limited in their effectiveness due in part to short circulation times and suboptimal biodistribution of the therapeutic enzymes. We previously engineered Chinese hamster ovary (CHO) cells to produce α-galactosidase A (GLA) with various N-glycan structures and demonstrated that elimination of mannose-6-phosphate (M6P) and conversion to homogeneous sialylated N-glycans prolonged circulation time and improved biodistribution of the enzyme following a single-dose infusion into Fabry mice. Here, we confirmed these findings using repeated infusions of the glycoengineered GLA into Fabry mice and further tested whether this glycoengineering approach, Long-Acting-GlycoDesign (LAGD), could be implemented on other lysosomal enzymes. LAGD-engineered CHO cells stably expressing a panel of lysosomal enzymes [aspartylglucosamine (AGA), beta-glucuronidase (GUSB), cathepsin D (CTSD), tripeptidyl peptidase (TPP1), alpha-glucosidase (GAA) or iduronate 2-sulfatase (IDS)] successfully converted all M6P-containing N-glycans to complex sialylated N-glycans. The resulting homogenous glycodesigns enabled glycoprotein profiling by native mass spectrometry. Notably, LAGD extended the plasma half-life of all three enzymes tested (GLA, GUSB, AGA) in wildtype mice. LAGD may be widely applicable to lysosomal replacement enzymes to improve their circulatory stability and therapeutic efficacy.

## 1 Introduction

Lysosomal storage diseases (LSDs) are a group of more than 60 inherited metabolic disorders caused by pathogenic mutations in genes encoding lysosomal proteins, leading to accumulation of undegraded substrates in the lysosome, and ultimately, cell death and impaired organ function (Neufeld, 1991; Platt et al., 2018). Currently, 15 enzyme replacement therapies (ERTs) are approved for the treatment of ten LSDs (Poswar et al., 2019), but challenges remain with regards to short half-life and limited delivery of replacement enzymes to hard-to-reach organs such as bone, kidney, heart, and brain (Desnick and Schuchman, 2012; Parenti et al., 2015). Cellular uptake of exogenously administrated lysosomal enzymes is thought to rely primarily on receptors that recognize specific N-glycan features of the therapeutic enzymes (Grubb et al., 2010). The main receptor for uptake of therapeutic lysosomal replacement enzymes with mannose-6-phosphate (M6P) N-glycans is the cation-independent M6P receptor (CI-MPR), which recycles to the cell surface and delivers enzymes to the lysosome (Chavez et al., 2007; Braulke and Bonifacino, 2009). Mannose receptors (MRs) are involved in uptake of lysosomal replacement enzymes with high-mannose N-glycans while the Ashwell-Morell receptor (AMR) (asialoglycoprotein receptor, ASGPR) binds N-glycans without sialic acid capping and exposed galactose residues and may also contribute to uptake of therapeutic glycoproteins (Stockert, 1995). Altering the glycan structures on lysosomal replacement enzymes, therefore, may influence their uptake, pharmacokinetics and biodistribution (Grubb et al., 2010; Tian et al., 2019).

Lysosomal enzymes undergo M6P-tagging of select N-glycans in the early Golgi apparatus, where the GlcNAc-1-phosphotransferase transfers GlcNAc-1-phosphate to specific mannose residues mainly on the α6 arm of high-Man N-glycans (Couso et al., 1986). Thereafter, the GlcNAc residue is removed by the uncovering N-acetylglucosamine-1-phosphodiester α-N-acetylglucosaminidase enzyme, generating the M6P modification (Mullis and Kornfeld, 1994). Recombinant lysosomal enzymes acquire different degrees of M6P on N-glycans at select N-glycosites (Dahms et al., 1989), and it has long been thought that increasing the M6P-tagging on N-glycans may improve uptake of lysosomal enzymes. For example, Alglucosidase alfa, a recombinant human acid α-glucosidase (rhGAA) enzyme used for treating glycogen storage disease type II (Pompe disease), has limited effect on clearance of glycogen in skeletal muscle cells and fails to improve the compromised autophagic flux. This has been considered to be due to low content of M6P moiety on rhGAA as well as low expression of CI-MPR on target muscle cells (Koeberl et al., 2011; Nascimbeni et al., 2012). Efforts to overcome these challenges include use of chemical conjugation of a synthetic oligosaccharide with M6P to increase content of M6P, which has resulted in improved uptake of rhGAA by muscle cells (Zhu et al., 2009; Zhou et al., 2011). One such engineered enzyme, Avalglucosidase alfa, was recently approved in the United States, while others are being evaluated in clinical trials (Dhillon, 2021). Alternatively, rhGAA expressed in moss and containing N-glycans with exposed terminal N-acetylglucosamine (βGlcNAc) residues has also resulted in increased uptake by differentiated myotubes with similar clearance of accumulated glycogen compared to Alglucosidase alfa (Hintze et al., 2020). MRs expressed on macrophages are considered to be the main receptors for uptake of recombinant β-glucocerebrosidase (GBA) used to treat Gaucher disease (Barton et al., 1991; Shaaltiel et al., 2007; Tekoah et al., 2013). Currently, three glycoengineered GBA enzymes (Imiglucerase, Velaglucerase alfa and Taliglucerase) with exposed mannose residues on N-glycans are used for treating non-neuropathic type I Gaucher disease. These were generated by employing exoglycosidase enzymes (Grabowski et al., 1995) or the mannosidase I inhibitor, kifunensine (Brumshtein et al., 2010), or by targeting to plant storage vacuoles using additional vacuolar sorting signal peptide (Shaaltiel et al., 2007) to increase terminal mannose residues and enhance cellular uptake.

As an alternative approach to modifying the N-glycan structure of the enzyme, complete elimination of interactions with glycan binding receptors has been explored to improve performance of lysosomal replacement enzymes. For example, chemical modification with sodium metaperiodate followed by borohydride reduction was employed to destroy glycan-binding of recombinant β-glucuronidase (GUSB) used for treatment of mucopolysaccharidosis (MPS) VII (Grubb et al., 2008). The chemically modified GUSB enzyme showed prolonged circulation and improved delivery to the brain, reducing neuronal substrate storage more effectively than native GUSB in a mouse model of MPS VII (Grubb et al., 2008). When the same approach was applied to recombinant sulfamidase and infused into a MPS IIIA mouse model, the chemically modified enzyme showed lower plasma clearance relative to the native enzyme and effectively reduced substrate accumulation in the brain (Gustavsson et al., 2019). While these studies are promising, it is currently unclear if this strategy of disrupting N-glycan interaction can be applied to other lysosomal enzymes to enhance their therapeutic performance. Notably, chemical modification of tripeptidyl peptidase 1 (TPP1) resulted in extended circulation time but did not improve targeting to the brain (Meng et al., 2012). In a study separate from the aforementioned, chemically modified sulfamidase was delivered to the brain in a MPS IIIA mouse model but central nervous system (CNS) substrate concentrations remained unchanged, presumably because the modified enzyme did not reach target cell types (i.e., neurons, glia) within the brain (Rozaklis et al., 2011).

**FIGURE 1:**
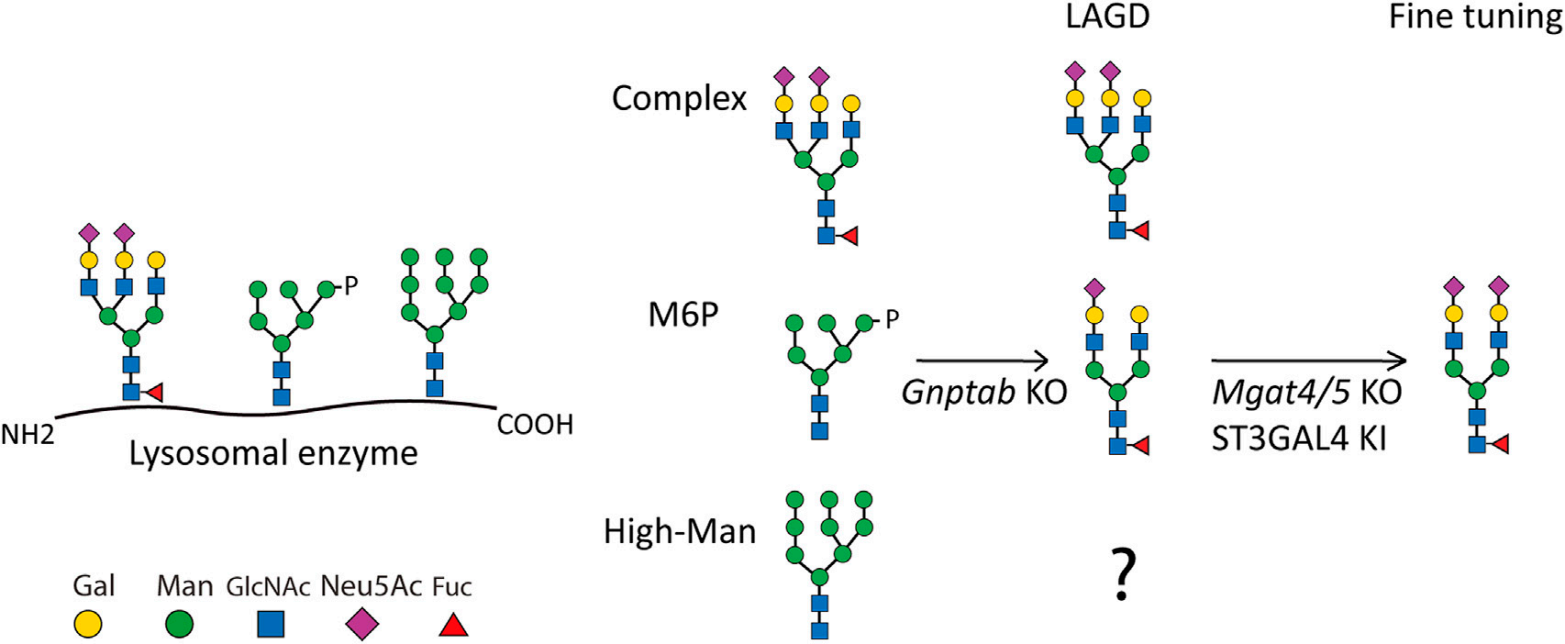
Depiction of the LAGD glycoengineering strategy for lysosomal enzymes. The three principle types of N-glycan structures are illustrated with the predicted outcomes indicated after KO of *Gnptab* in CHO cells. Further fine-tuning steps to obtain homogeneous complex biantennary N-glycans with α2-3 sialic acid capping are shown (Tian et al., 2019). Glycan symbols according to Symbol Nomenclature for Glycans format. Gal denotes galactose, Man denotes mannose, GlcNAc denotes N-Acetylglucosamine, Neu5Ac denotes N-Acetylneuraminic acid, Fuc denotes fucose, and P denotes phosphate.

We previously developed a glycoengineering design, coined Long-Acting-GlycoDesign (LAGD), in Chinese hamster ovary (CHO) cells to produce α-galactosidase A (GLA) enzyme lacking M6P (Tian et al., 2019). The LAGD engineering design results in conversion of the normally M6P-tagged N-glycans into complex type N-glycans capped with sialic acids (Figure 1). GLA deficiency leads to Fabry disease, a X-linked lysosomal storage disorder in which the enzyme’s major substrate, globotriasylceramide (Gb3), progressively and preferentially accumulates in lysosomes of renal cells and cardiomyocytes. Currently, two rhGLA enzymes (Fabrazyme and Replagal) are approved for the treatment of this disease. We demonstrated that elimination of M6P on N-glycans did not impair cellular uptake of the enzyme and that the LAGD engineered GLA (GLA LAGD) cleared Gb3 as effectively as Fabrazyme in key target organs of Fabry disease mice using a single dosing regimen (Tian et al., 2019). Notably, plasma half-life of GLA LAGD was significantly extended and biodistribution was improved compared to Fabrazyme, with reduced uptake by liver and increased uptake in heart.

Here, we extended our studies by evaluating the effects of repeated infusions of GLA LAGD in Fabry mice. We further explored whether LAGD engineering is applicable to other lysosomal enzymes with different numbers and structures of N-glycans, using glycosylasparaginase (AGA), GUSB, cathepsin D (CTSD), TPP1, acid α-glucosidase (GAA), and iduronate-2-sulfatase (IDS) as model enzymes. We demonstrate that the LAGD engineering design consistently resulted in conversion of M6P-tagged N-glycans to complex-type with sialic acid capping and that the more homogeneous LAGD glycodesign reduced the glycoproteoform variability and facilitated direct analysis of the therapeutic glycoproteins by high-resolution native mass spectrometry (MS) (Leney and Heck, 2017; Tamara et al., 2020). Importantly, LAGD engineering extended circulation times of the enzymes when administered to wildtype mice.

## 2 Materials and methods

### 2.1 Single and repeated dosing studies in Fabry male mice

Fabry mice, generated as previously described (Denis et al., 1975) and backcrossed onto the C57BL/6 background, were bred and maintained in a barrier facility at the Icahn School of Medicine at Mount Sinai (ISMMS). All animal procedures were reviewed and approved by the ISMMS Institutional Animal Care and Use Committee. Eight to 12 week old male Fabry mice were intravenously infused with either GLA LAGD or Fabrazyme (0.3 or 1.0 mg/kg) or saline (i.e., controls). For repeated dosing studies, mice were infused at 0, 2, 4, 6, and 8 weeks. Enzymes were diluted in saline so that each mouse received an injection volume of 0.01 mL/g weight per dose. For both single and repeated dosing studies, mice were euthanized 24 h after the last infusion, by deeply anesthetizing and perfusing with saline *via* the left cardiac ventricle. Tissues were harvested and stored at −80°C until anaylsis.

### 2.2 Lipid analysis in organs

Frozen organs were thawed and 20–50 mg of tissue was homogenized using a Tissuelizer (Qiagen) with two beads at 30 Hz for 2 min following addition of 500 µL dH_2_O. Homogenates were transferred to a new tube. The emptied homogenization tubes were washed with 500 µL of dH_2_O and vortexed briefly, and the contents were added to the respective homogenates. 20 μL of homogenized kidney, liver and heart were mixed with 40, 60, and 20 µL of dH_2_O, respectively, and vortexed. These diluted homogenate samples were used for bicinchoninic acid (BCA) analysis. Lipid extraction was performed on 20 µg of protein of kidney and liver and 50 µg of protein of heart homogenized tissues. Lipid extraction was performed as described previously with minor modifications (Sampaio et al., 2011). In brief, 150 mM ammonium bicarbonate solution was added to the homogenate samples to make 200 µL total aqueous solution. 1 mL of Chl:MeOH (10:1 v/v, pre-mixed with an internal standard mixture composed of 500 pmol of cholesterol (Chol)-d6, 100 pmol of cholesterol ester (CE)-d7 16:0, 100 pmol of diacylglycerol (DAG) 17:0/17:0, 50 pmol of triacylglycerol (TAG) 17:0/17:0/17:0, 100 pmol of sphingomyelin (SM) d18:1/12:0, 30 pmol of ceramide (Cer) d18:1/12:0, 30 pmol of galactosyl ceramide (GalCer) d18:1/12:0, 50 pmol of lactosyl ceramide (LacCer) d18:1/12:0, 300 pmol of phosphatidylcholine (PC) 17:0/17:0, 50 pmol of phosphatidylethanolamine (PE) 17:0/17:0, 30 pmol of phosphatidylinositol (PI) 16:0/16:0, 50 pmol of phosphatidylserine (PS) 17:0/17:0, 30 pmol of phosphatidylglycerol (PG) 17:0/17:0, 30 pmol of phosphatic acid (PA) 17:0/17:0, 25 pmol of globotriaosylceramide (Gb3) d18:1/17:0, 25 pmol of globotriaosylsphingosine (lysoGb3) d18:1-d7, 25 pmol of ganglioside M3 (GM3) d18:1-18:0d5, 25 pmol of Ganglioside M1 (GM1) d18:1-17:0 and 25 pmol of ganglioside M2 (GM2) d20:1-17:0) was mixed with the samples and vortexed at 1,100 rpm in an Eppendorf shaker for 2 h at 4°C. After short centrifugation, the lower organic phase was collected and dried by speedvac. The remaining aqueous phase was reextracted by addition of 1 mL of Chl:MeOH 2:1 and vortexed at 1,100 rpm in an Eppendorf shaker for 1 h at 4°C. After short centrifugation, the lower organic phase was collected and dried by speedvac. Both lipid extracts were dissolved in 100 µL of infusion mixture consisting of 7.5 mM ammonium acetate dissolved in propanol:chloroform:methanol [4:1:2 (vol/vol)].

Samples were analysed by direct infusion in a QExactive Plus mass spectrometer (Thermo Fisher Scientific) equipped with a TriVersa NanoMate ion source (Advion Biosciences) with minor modifications (Surma et al., 2015). In brief, 5 μL of sample was infused with gas pressure and voltage set to 1.25 psi and 0.95 kV, respectively. DAG, TAG and CE were detected as ammonium adducts and PC, PC O- and SM were detected as protonated ions in the 10:1 extract, by positive ion mode fourier transform mass spectrometry (FTMS) by scanning m/z = 580–1,000 Da, Rm/z = 200 = 280,000 with lock mass activated at a common background (m/z = 680.4802) for 30 s. Every scan is the average of two micro-scans, automatic gain control (AGC) was set to 1E6 and maximum ion injection time (IT) was set to 200 ms. Cer and hexosylceramide (HexCer) were detected as acetate adducts and PE, PE O-, PG were detected as deprotonated ions in the 10:1 extract, after polarity switch by negative ion mode FTMS by scanning m/z = 520–1,050 Da, at Rm/z = 200 = 280,000 with lock mass activated at a common background (m/z = 529.4626) for 30 s. Every scan is the average of two microscans, AGC was set to 1E6 and IT was set to 50 ms. Hex2Cer, lysoGb3 and Gb3 were detected as protonated ions in the 2:1 extract in positive ion mode FTMS by scanning m/z = 750–1,600 Da, at Rm/z = 200 = 280,000 with lock mass activated at a common background (m/z = 1,194.8179) for 30 s. GM1, GM2, and GM3 were detected as deprotonated ions in the 2:1 extract in negative ion mode after polarity switch in FTMS by scanning m/z = 1,100–1,650 Da, at Rm/z = 200 = 280,000 with lock mass activated at a common background (m/z = 1,175.7768) for 30 s. Every scan is the average of two micro-scans, AGC was set to 1E6 and IT was set to 50 ms. PA, PI and PS were detected as deprotonated ions in the 2:1 extract in negative ion mode in FTMS by scanning m/z = 520–1,100 Da, at Rm/z = 200 = 280,000 with lock mass activated at a common background (m/z = 529.4626) for 30 s. Every scan is the average of two micro-scans, AGC was set to 1E6 and IT was set to 50 ms. All data were acquired in centroid mode.

### 2.3 Stable expression of recombinant human AGA, GUSB, CTSD, TPP1, GAA and IDS in CHO cells

CHOZN GS−/− cells (Merck) were maintained in suspension cultures in serum-free media (EX-CELL CHO CD Fusion, Merck) supplemented with 4 mM L-glutamine, as previously described (Tian et al., 2019). An expression construct containing the entire coding sequence of human *AGA* was synthesized by Genewiz, United States. Full-length cDNA of human *GUSB*, *TPP1*, and *GAA* were purchased from Horizon Discovery, United Kingdom, while human *IDS* cDNA was purchased from Sino Biological. C-terminal His-tagged CTSD was produced as previously reported (Marques et al., 2020). All reporter constructs were cloned into pCGS3 (Merck). Cells were seeded at a density of 0.5 × 10^6^ cells/mL in T25 flasks (NUNC) 24 h prior to transfection. Approximately 2 × 10^6^ cells were transfected with 8 μg endotoxin-free plasmids using Amaxa kit V and program U24 with Amaxa Nucleofector 2B (Lonza). 72 h post-transfection, cells were plated at 500–1,000 cells/well in 96-wells in 200 μL Minipool Plating Medium containing 80% EX-CELL^®^ CHO Cloning Medium (Merck) and 20% EX-CELL CD CHO Fusion media without glutamine for selection. Screening of high expression minipools were performed by determining enzyme activity in spent media for AGA, GUSB, GAA and IDS, by SDS-PAGE for TPP1 and CTSD. Selected minipools were further single cell sorted by fluorescence-activated cell sorting (FACS) (Sony) and expanded in 50 mL TPP TubeSpin^®^ shaking Bioreactors (180 rpm, 37°C and 5% CO_2_).

### 2.4 CRISPR/Cas9 targeted KO in CHO cells

Knockout (KO) of GlcNAc-1-phosphate transferase subunits alpha and beta (*Gnptab*) gene was carried out in selected CHO clones that stably express the lysosomal enzymes of interest. Cells were seeded at a density of 0.5 × 10^6^ cells/mL in T25 flasks 24 h prior to transfection, and ∼2 × 10^6^ cells and 1 μg each of plasmid DNA of Cas9-GFP and gRNA were used for electroporation. 48 h after electroporation, cells with GFP expression were enriched by FACS. After culturing for 1 week, cells were single cell sorted by FACS into 96-wells. KO clones with desired mutations were identified using a rapid and efficient screening method, Indel Detection by Amplicon Analysis (IDAA), as previously described (Yang et al., 2015a). Final clones were verified by Sanger sequencing. On average 2–5 clones with frameshift mutations were selected from each targeting event. The full list of CRISPR gRNA design and PCR primers used is listed elsewhere (Tian et al., 2019).

### 2.5 Zinc finger nuclease-mediated gene knockin into CHO cells

Knockin (KI) of human ST3 beta-galactoside alpha-2,3-sialyltransferase 4 (*ST3GAL4*) and the human sulfatase modifying factor 1 (*SUMF1*) cDNA with a 3′-terminal HA tag sequence into AGA- and IDS-expressing cell lines, respectively, targeting site-specific CHO safe-harbor locus was performed based on the ObLiGaRe strategy (Maresca et al., 2013). In brief, ∼2 × 10^6^ cells were transfected with 2 µg of each zinc finger nuclease (ZFN; Merck) tagged with GFP/Crimson reporter genes and 5 µg of EPB69 donor plasmid containing inverted CHO safe-harbor locus ZFN binding sites which flanked a cassette that included the CMV promoter, followed by the *ST3GAL4* or *SUMF1* cDNA and a bovine growth hormone polyadenylation terminator. KI clones were first screened by immunocytology using Maackia Amurensis Lectin I, Biotinylated (Vector labs) and Streptavidin Alexa Fluor 488 conjugate antibodies (Invitrogen) for ST3GAL4 and HA probe antibody (Santa Cruz Biotechnology) and polyclonal rabbit anti mouse, FITC antibody (Dako) for SUMF1. Positive clones were further characterized by PCR, using primers specific for the junction area between the donor plasmid and the safe-harbor locus.

### 2.6 Purification of recombinant expressed AGA, GUSB, CTSD, TPP1, GAA and IDS

Culture media were centrifuged at 500 × g for 20 min and filtered (0.45 μm). For AGA, 20% v/v of conditioning buffer (70 mM Tris-HCl, pH 7.0) was added to the media and loaded on column packed with Q-FastFlow Sepharose (GE Healthcare) pre-equilibrated with 5 column volume (CV) equilibration buffer (20 mM Tris-HCl, 20 mM sodium acetate, 70 mM sodium chloride, pH 6.8). After washing the column with 6 CV of wash buffer (20 mM Tris-HCl, 20 mM sodium acetate, 70 mM sodium chloride, pH 6.8), the enzyme was one-step eluted with elution buffer (25 mM sodium acetate, 250 mM NaCl, pH 4.5) into a tube containing 300 mM sodium phosphate (pH 7.3). The eluates were diluted with 50% v/v of 4 M (NH_4_)_2_SO_4_ and further loaded on a Phenyl-Sepharose Fast Flow (high substitution) column (GE Healthcare). After washing and equilibrating the column with 5 CV of 2 M (NH_4_)_2_SO_4_, 20 mM Tris-HCl, pH 7.0, the enzyme was eluted with elution buffer in gradient (2–0 M (NH_4_)_2_SO_4_, 20 mM Tris-HCl, pH 7.0). For GUSB, medium was diluted 3-fold with conditioning buffer (10 mM Tris-HCl, 1 mM β-glycerophosphate, pH 8.0) and loaded on HiTrap DEAE Sepharose Fast Flow column (GE Healthcare) pre-equilibrated with 2 CV conditioning buffer. After washing the column with wash buffer (10 mM Tris-HCl, 1 mM β-glycerophosphate, 50 mM NaCl, pH 8.0), the enzyme was eluted in elution buffer (10 mM Tris-HCl, 1 mM β-glycerophosphate, 300 mM NaCl, pH 8.0). Eluates were diluted in 3-fold volume of conditioning buffer (10 mM Tris-HCl, 1 mM β-glycerophosphate, pH 8.0) and loaded on Mono-Q column (GE Healthcare) and eluted with elution buffer in gradient (0–1 M NaCl, 10 mM Tris-HCl, 1 mM β-glycerophosphate, pH 8.0). For CTSD, medium was diluted 3:1 (v/v) with conditioning buffer (100 mM Tris, 40 mM imidazole 1.2 M NaCl, pH 8.0) and loaded on a 1 mL packed Histrap column (GE Healthcare) pre-equilibrated with 5 CV of conditioning buffer (25 mM Tris, 10 mM imidazole, 300 mM NaCl, pH 8.0). The column was washed with 5CV of conditioning buffer and the enzyme was eluted with 4CV of elution buffer (250 mM imidazole, 25 mM Tris, 300 mM NaCl, pH8.0). For TPP1, medium was diluted 3:1 (v/v) with conditioning buffer (20 mM Tris–HCl, pH 7.6) and loaded on DEAE-Sepharose Fast Flow column pre-equilibrated with 5 CV of conditioning buffer. The enzyme was eluted stepwise with 25, 100, 200 mM, 400 mM NaCl in 2 CV of conditioning buffer. Eluates were diluted with 3X volume of conditioning buffer and thereafter loaded onto Mono-Q column and eluted with gradient NaCl (0-1 M NaCl, 20 mM Tris–HCl, pH 7.6). For GAA, medium was dialysed over night and loaded on a DEAE-Sepharose Fast Flow column pre-equilibrated with 5 CV of 25 mM MES, pH 6.5. The enzyme was eluted in one step with 200 mM NaCl in 2 CV of 25 mM MES, pH 6.5. Eluates were adjusted to 1 M (NH_4_)_2_SO_4_ and loaded on a Phenyl-Sepharose Fast Flow column pre-equilibrated with 25 mM MES, 1 M (NH_4_)_2_SO_4,_ pH 6,5. The enzyme was eluted one step at 50 mM (NH_4_)_2_SO_4_ in 25 mM MES. The eluates were buffer exchanged and further loaded on Mono-Q column and eluted in gradient in 0–1 M NaCl, 25 mM MES, pH 6.5. For IDS, medium was dialysed overnight and loaded on a DEAE-Sepharose Fast Flow column pre-equilibrated with 5 CV of 25 mM MES, pH 6.5. The enzyme was eluted one step with 200 mM NaCl in 2 CV of 25 mM MES, pH 6.5. Eluates were adjusted to 2 M NaCl and loaded on a Phenyl-Sepharose Fast Flow column pre-equilibrated with 25 mM MES, 2 M NaCl, pH 6,5. The enzyme was eluted one step at 150 mM NaCl in 25 mM MES, pH 6,5. The eluates were buffer exchanged and further loaded on Mono-Q column and eluted in gradient in 0–1 M NaCl, 25 mM MES, pH 6.5.

### 2.7 Enzyme activity assays

GLA activities were determined in Fabry mouse tissues using previously described methods (Desnick et al., 1973). In brief, tissue samples were homogenized in chilled reporter lysis buffer (Promega) and protease inhibitor (Pierce) was added to the lysates. Protein concentrations were determined using the Bio-Rad Colorimetric Protein Assay Kit. 10 μL of tissue lysate was added to an equal volume of 10 mM 4-methylumbelliferyl-α-D-galactopyranoside (Sigma-Aldrich), dissolved in assay buffer (0.2 M citrate, 0.4 M phosphate buffer, pH 4.4), and 0.1 M N-acetylgalactosamine (Sigma Aldrich), the latter to inhibit α-galactosidase B activity (Mayes et al., 1981). Following a 30 min incubation at 37°C, reactions were terminated by the addition of 480 μL of 0.1 M ethylenediamine, pH 10.3. The amount of 4-methylumbelliferone (4-MU) produced was determined by measuring fluorescence using a Synergy H1 fluorometer (BioTek). Tissue α-Gal A activities were expressed as nmol of 4-MU produced per h per mg of total protein (nmol/h/mg). Measurement of plasma GLA activities in wildtype mice for PK studies was performed as described above with the following modifications: lysates were incubated with 5 mM 4-methylumbelliferyl α-D-galactopyranoside in assay buffer [20 mM citrate, 30 mM sodium phosphate (pH 4.4), 0.1 M N-acetylgalactosamine, and 4 mg/mL BSA], and the reaction was stopped by addition of stop buffer (0.1 M Glycine, 0.1 N NaOH], as previously described (Shen et al., 2016).

AGA activity was measured with 1 mM L-aspartic acid β-(7- amido-4-methylcoumarin) in 10% SuperBlock and 90% 50 mM Tris-HC (pH 7.5) for 60 min at 37°C, and then adding 100 µL of stop buffer [0.2 M glycine, 0.175 M NaOH (pH 10.6)], as previously described (Mononen et al., 1993). GUSB enzyme assay was performed using 10 mM 4-methylumbelliferyl-β-D-glucuronide (Merck) in 0.1 M sodium acetate (pH 4.6) at 37°C for 30 min, and reactions were stopped by 0.1 M sodium carbonate (Grubb et al., 2008). GAA activity assay was performed with 3 mM 4-methylumbelliferyl-a-D-glucopyranoside (Merck) in assay buffer (30 mM sodium citrate, 40 mM sodium phosphate dibasic, pH 4.0) at 37°C for 3 h (Flanagan et al., 2009). Reactions were stopped by the addition of an equal volume of 0.4 M glycine, pH 10.8. IDS activity assay was performed with 2.5 mM 4-Methylumbelliferyl sulfate potassium salt (Merck) in 50 mM sodium acetate, at 37°C for 4 h (Dean et al., 2006). Reactions were stopped with glycine carbonate buffer (pH 10.7). Fluorescence was measured by microplate reader with 360/40 nm excitation and 440/30 nm emission filters.

### 2.8 Sample preparation for released N-glycans analysis

Release of N-glycan, Rapiflour labeling and purification of Rapiflour labeled N-glycan were performed according to the manufactures’ protocols. Briefly, 15 µg of each enzyme was heat denatured at 90°C for 3 min in 6 µL of buffer solution containing 5% (w/v) RapiGest SF and 18.2 megohm water. After cooling down to room temperature enzymes were deglycosylated with 1.2 µL of RapiPNGase F at 50°C for 5 min. Thereafter, the enzymes were labeled with 12 µL of the RapiFlour-MS Reagent Solution at room temperature for 5 min and 358 µL of acetonitrile solution was added to dilute the reaction. To enrich the glycans, hydrophilic interaction liquid chromatography solid phase extraction (HILIC SPE) was performed by Waters GlycoWorks HILIC µElution Plate. The plate was washed with 200 µL of Milli-Q water, followed by equilibration with 200 µL of 15:85 water/acetonitrile. After loading the acetonitrile-diluted sample, the well was washed twice with 600 µL of 1:9:90 (v/v/v) formic acid/water/acetonitrile. The glycans were eluted with 30 µL of GlycoWorks SPE Elution Buffer (200 mM ammonium acetate in 5% acetonitrile).

### 2.9 Sample preparation for N-glycopeptides site specific analysis

25 µg of purified AGA, GUSB CTSD, and GAA were dissolved in 50 mM ammonium bicarbonate (AmBic) buffer (pH 7.4) and further reduced with 10 mM dithiothreitol (DTT) at 60°C for 45 min on shaker, followed by alkylation with 20 mM iodoacetamide (IAA) at 25°C for 30 min in darkness. AGA, GUSB, CTSD were subjected to proteolytic digestion with chymotrypsin (1:40 enzyme-substrate ratio), while GAA was digested in gel with trypsin (1:25 enzyme-substrate ratio) after SDS-PAGE separation. The reaction was quenched with 1 µL trifluoroacetic acid (TFA) and the digested sample was desalted by custom-made modified StageTip colums with three layers of C18 and two layers of C8 membrane (3 M Empore disks, Sigma-Aldrich). Samples were eluted with two steps of 50 µL 50% methanol in 0.1% formic acid. Final sample was aliqoted in two equal parts. The first aliquot was placed into a glass insert (Agilent), dried completely in SpeedVac (Eppendorf) and further re-dissolved in 50 µL 0.1% formic acid (FA) and submitted for nLC-MS analysis. The second aliqout was placed inside an Eppendorf tube, dried completely using SpeedVac, and then re-dissolved in 50 µL of 50 mM AmBic buffer (pH 7.4) and incubated with PNGase F (1U per sample) for 12 h with shaking at 37°C. Samples treated with PNGase F were desalted and dried using the same methods mentioned above for the first aliqout and submitted for nLC-MS/MS analysis.

### 2.10 nLC-MS/MS analysis for glycan and glycopeptide analysis

An EASY-nLC 1000 LC system (Thermo Fisher Scientific) interfaced *via* nanoSpray Flex ion source to an Orbitrap Fusion Lumos MS (Thermo Fisher Scientific) was used for MS and MS/MS analyses. A single analytical column setup using PicoFrit Emitters (New Objectives, 75 µm inner diameter) custom packed with Reprosil-Pure-AQ C18 phase (Dr. Maisch, 1.9-µm particle size, 19–21 cm column length) was applied in nLC. 2 μL of each sample was injected onto the column, followed by elution with a gradient of Solvent B from 2% to 25% at 200 nL/min for 45 min (Solvent A: 100% H_2_O+ 0.1% (v/v) formic acid; Solvent B: 100% acetonitrile +0.1% (v/v) formic acid). With the nominal resolution setting of 120,000, precursors of MS1 scan (m/z 350-2,000) were obtained. Then HCD-MS2 of the five most abundant multiply charged precursors in the MS1 spectrum was acquired at the nominal resolution setting of 60,000. To trigger data-dependent fragmentation events, the minimum MS1 signal threshold was 50,000. Targeted MS/MS analysis was performed by setting up a targeted MSn (tMSn) Scan Properties panel. 30 targeted entries were included in the Mass List Table.

### 2.11 Sample preparation and analysis for native MS

Purified recombinant proteins were lyophilized and re-dissolved in 150 mM aqueous ammonium acetate (pH 7.5) with ultrafiltration by a 10 kDa cut-off filter (Sartorius Stedim Biotech, Germany). Some aliquots of samples were treated with 0.02 U of neuraminidase (Roche, IN, United States), either by itself or in combination with phosphatase (Sigma), and incubated at room temperature overnight. Samples were analyzed on a modified Exactive Plus Orbitrap instrument with ultra-high mass range, as described (Čaval et al., 2018). Briefly, spray voltage was set to 1.2–1.3 V, source fragmentation and collision energy were set in the range of 15%–25% to achieve optimal desolvation. The resolution was set to 25,000 at m/z 200.

### 2.12 Data analyses

#### 2.12.1 N-glycan and N-glycopeptide site specific compositional analysis

Glycopeptide compositional analysis was performed from *m/z* features using in-house written SysBioWare software (Vakhrushev et al., 2009). For *m/z* feature recognition from full MS scans Minora Feature Detector Node of the Proteome discoverer 2.3 (ThermoFisher Scientific) was used. A list of precursor ions (*m/z*, charge and retention time) was imported as ASCII data into SysBioWare and compositional assignment within 5 ppm mass tolerance was performed. The main building blocks used for the compositional analysis were: NeuAc, Hex, HexNAc, dHex and phosphate. The most prominent peptides corresponding to each potential glycosite were determined experimentally by comparing the yield of deamidated peptides before and after PNGase F treatment. The peptide sequence was determined by HCD MS/MS and the abundance level was calculated from PD 2.3. For N-glycopeptide compositional analysis the corresponding peptides were also added as building blocks.

A list of potential glycan and glycopeptides for each glycosite was generated and the top 10–15 of the most abundant candidates were selected for targeted MS/MS analysis to confirm the proposed structure. Each targeted MS/MS spectrum was subjected to manual interpretation. Since the same N-glycan composition may represent various isobaric structures, the final glycan structures were proposed according to literature data, predicted enzyme functions of the targeted genes, along with information in MS/MS fragments.

#### 2.12.2 Native MS deconvolution

For intact mass analysis, raw spectra were deconvoluted to zero-charge by Intact Mass software (Protein Metrics, CA, United States) using default settings (Bern et al., 2018). Glycoproteoforms were annotated by in-house written SysBioWare software (Vakhrushev et al., 2009) using average masses of hexose, N-acetylhexosamine, and the known backbone mass of the naked protein sequence increment (GLA and AGA).

#### 2.12.3 Lipid analysis

All data were analysed with the lipid identification software, LipidXplorer (Herzog et al., 2011). Tolerance for MS identification was set to 2 ppm. Data post-processing and normalization to internal standards were done manually.

### 2.13 Pharmacokinetic evaluation in wildtype mice

All animal procedures were reviewed and approved by The Danish Animal Experiments Inspectorate. Purified enzymes were injected into eight to 12 week old female balbc/A wildtype mice *via* the tail-vein at a dose of 1 mg/kg body weight at a concentration of 147 μg/mL. An average volume of 150 µL was infused. Blood samples were collected by cheek bleed at four different time points. Plasma was separated by centrifugation and 2.5 µL of plasma was used for enzyme activity assay.

## 3 Results

### 3.1 The Long-Acting-GlycoDesign (LAGD) genetic glycoengineering design

The LAGD glycoengineering design involves knock out (KO) of *Gnptab* to eliminate M6P-tagging (Tian et al., 2019) (Figure 1). The LAGD design may be combined with further engineering to improve homogeneity by reducing N-glycan branching to biantennary structures and enhancing sialylation if needed (Tian et al., 2019). GNPTAB transfers GlcNAc-1-phosphate to select N-glycan positions on lysosomal glycoproteins, and other N-glycans that are not modified are generally coverted to complex N-glycans. N-glycoproteins including lysosomal enzymes may also contain N-glycans at specific positions that are maintained as high-mannose (Man) N-glycans following trafficking of the secretory pathway. These high-Man N-glycans resilient to the glycosylation maturation processes are not predicted to be affected by the LAGD glycoengineering strategy, and hence expected to remain on the secreted enzyme protein (Figure 1). To the best of our knowledge, the role of such retained high-Man N-glycans has not been experimentally addressed.

### 3.2 Repeat infusion of LAGD engineered GLA in a Fabry mouse model

**FIGURE 2:**
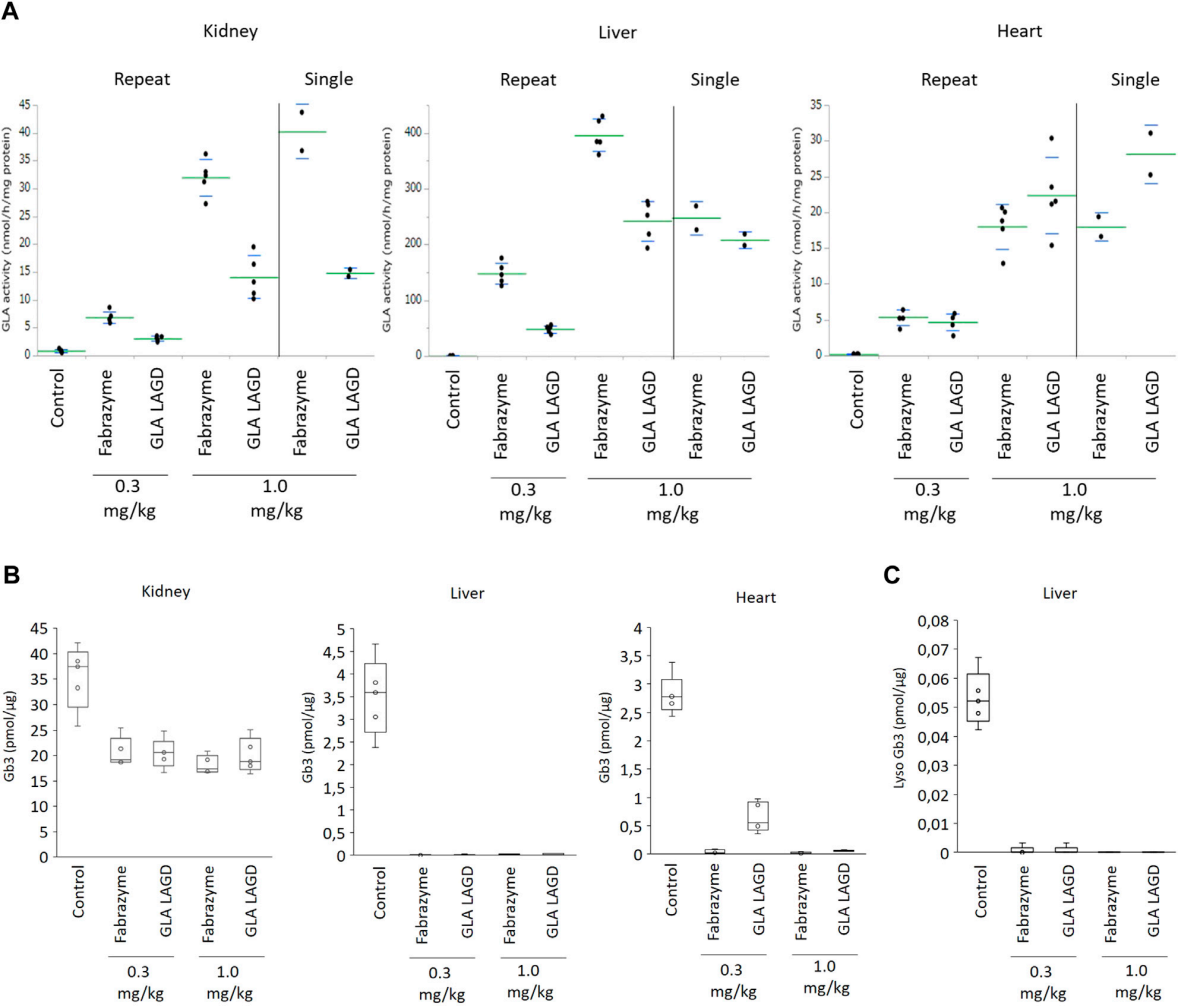
GLA activities and Gb3 analysis in repeated dose study in Fabry mouse model. **(A)** GLA enzyme activity represented as relative fluorescence intensity in indicated organs of mice after infusion of 0.3 or 1.0 mg/kg Fabrazyme or GLA LAGD, or saline control (n = 5 in each group) every 2 weeks for 5 times. Included are data of single infusion studies at 1.0 mg/kg that were repeated to confirm findings of our previous study (Tian et al., 2019). **(B)** Gb3 substrate level quantified by shotgun lipidomics MS analysis of the organs of mice mentioned above. **(C)** Lyso Gb3 level in liver of mice mentioned above quantified by shotgun lipidomics MS analysis.

The GLA protein contains three N-glycans of which two are M6P-tagged and one matured to complex-type, and the LAGD engineering converts all three sites to homogeneous complex-type N-glycans (Tian et al., 2019). We previously showed that plasma half-life of LAGD-engineered GLA with homogeneous biantennary N-glycans capped with α2-3 sialic acid (GLA^KO *Gnptab/g/Mgat4b/5* KI *ST3GAL4*^) was significantly extended and biodistribution was improved compared to Fabrazyme in a single dose study in Fabry mice. Here, we extended our studies by repeated infusion study. Fabry mice were infused with LAGD engineered GLA (Tian et al., 2019) or Fabrazyme at a dose of 0.3 or 1 mg/kg every 2 weeks for a total of five infusions. When assessed 24 h after the last infusion, 1.0 mg/kg GLA LAGD resulted in mean GLA activities of 14 ± 4, 243 ± 36 and 22 ± 5 nmol/h/mg in kidney, liver and heart, respectively, whereas the same dose of Fabrazyme resulted in 32 ± 3, 396 ± 29 and 18 ± 3 nmol/h/mg, respectively (Figure 2A). These findings were consistent with those of our previous single dose study and confirmed that GLA LAGD resulted in lower GLA activities in liver and kidney and higher cardiac activity compared to Fabrazyme (Figure 2A) (Tian et al., 2019). At the 0.3 mg/kg dose, mean GLA activities were 3.1 ± 0.4, 48 ± 7 and 4.7 ± 1.2 nmol/h/mg in kidney, liver and heart, respectively, for GLA LAGD-infused mice, while they were 6.9 ± 1.1, 148 ± 20 and 5.4 ± 1.1 nmol/h/mg in Fabrazyme-infused mice (Figure 2A). Thus, at the lower dose, GLA LAGD led to slightly lower enzymatic activity in heart compared to Fabrazyme, whereas in liver and kidney, the patterns in activities were similar to those at 1.0 mg/kg.

We then evaluated the effects of repeated GLA LAGD dosing on clearance of accumulated Gb3 in these three organs using a shotgun lipidomics approach that allows not only the quantification of Gb3 but also the comprehensive and quantitative profiling of the organs lipidome (Sampaio et al., 2011). GLA LAGD and Fabrazyme, at both 0.3 mg and 1 mg/kg doses, completely cleared Gb3 and its deacylated derivative, lysoGb3 in liver, while partial reduction of Gb3 levels was achieved in kidney (Figures 2B, C), consistent with previous single-dose studies (Tian et al., 2019). Remarkably, not all the Gb3 isoforms present in the kidney were impacted at the same extent by the enzyme treatments (Supplementary Figure S1A). Sorting the individual identified Gb3 isoforms by chain length, unsaturation and hydroxylation level (Supplementary Figures S1B–D), it became apparent that mono- and di-hydroxylated Gb3 isoforms are not affected by the enzyme treatment (Supplementary Figure S1B), and these glycolipid species are found only in kidney (Supplementary Figures S1B–D). These findings may explain the higher residual Gb3 found after enzyme treatment in the kidney relative to the other tested organs. Mean residual Gb3 concentrations in kidney were comparable between mice treated with Fabrazyme and GLA LAGD, both at 0.3 and 1.0 mg/kg, and ranged between 51%–58% of saline-treated Fabry controls. In the heart, 1.0 mg/kg GLA LAGD and Fabrazyme both reduced Gb3 concentrations to <3% of saline controls, but at the 0.3 mg/kg dose, GLA LAGD-infused mice still retained 23% of controls while Fabrazyme-infused mice had <2% of controls (Figure 2B). Comparing the lipidome profiles of the different organs revealed little or no changes in other lipid classes (Supplementary Figures S2A–C), in agreement with the restricted substrate specificity of GLA. The only changes observed were in liver, where some phospholipids (PCO-, PE and PS) were slightly decreased. Together, these findings show that repeated dosing of GLA LAGD leads to efficient enzyme uptake and effective substrate clearance in key organs despite the complete lack of M6P moieties. GLA LAGD resulted in enhanced targeting to the heart compared to Fabrazyme at 1.0 mg/kg, the ‘standard’ therapeutic dose, although this effect was lost at the lower 0.3 mg/kg dose.

### 3.3 The LAGD glycoengineering design is applicable to other lysosomal enzymes

**FIGURE 3:**
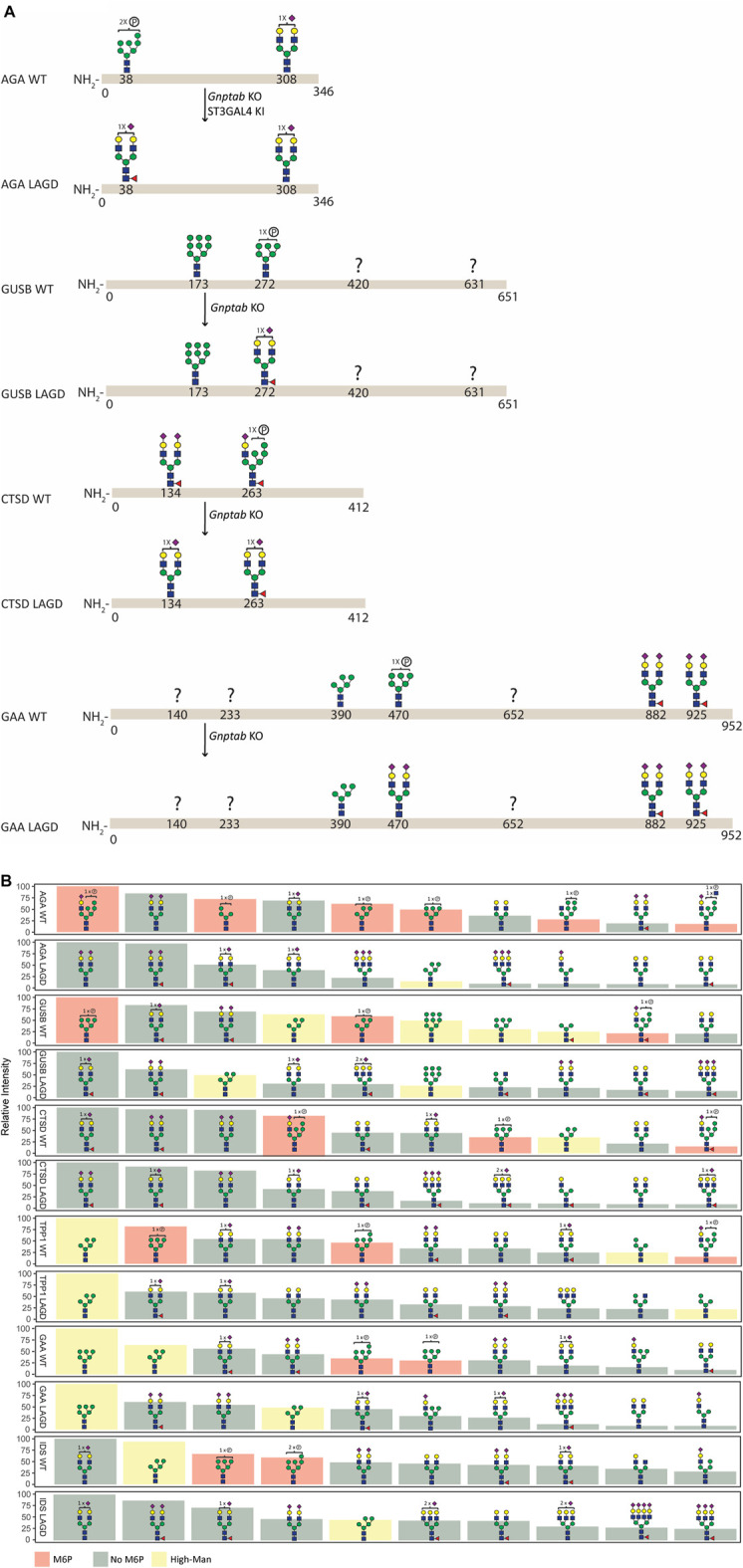
N-glycan analysis of purified recombinant lysosomal enzymes (AGA, GUSB, CTSD, TPP1, GAA, and IDS) produced in CHO^WT^ and engineered CHO cells. **(A)** Summary of site-specific N-glycopeptide analysis of purified recombinant lysosomal enzymes (AGA, GUSB, CTSD and GAA) produced in CHO^WT^ and engineered CHO cells. The most abundant glycan structure at N-glycosites of each enzyme produced in CHO^WT^ and engineered CHO clones are displayed on the top and bottom panels, respectively, with arrows indicating genetic editing strategy. Question mark indicates that no site-specific glycan structure was obtained for the partucular site. All glycan structures at each glycosite were confirmed by tandem mass spectrometry (MS/MS) analysis. **(B)** Summary of released *Rapi*Fluor-labeled N-glycan profiling showing the ten most abundant predicted species (normalized to most abundant structure).

To validate the LAGD glycoengineering design more generally for lysosomal enzymes, we first established CHOZN GS−/− cell lines stably expressing AGA, GUSB, CTSD, TPP1, GAA, or IDS, which were designated as wild type (CHO^WT^) cells (Table 1). LAGD design was then introduced into each of the established CHO clones by CRISPR/Cas9-mediated KO of *Gnptab* using a validated gRNA (Tian et al., 2019). In the case of AGA, we also introduced the human *ST3GAL4* gene by site-directed knock-in (KI) using ZFNs. To enhance the activity of IDS we introduced *SUMF1*, which encodes an enzyme that modifies the catalytic residue of IDS and is essential for its catalytic activity (Fraldi et al., 2007), by site-specific KI. CHO^WT^ and the LAGD-engineered CHO clones produced approximately the same amounts of expressed protein with final purified yields of 20–50 mg/L. Viability, growth, and productivity of the engineered cell clones were not affected by gene targeting. Secreted enzymes from CHO^WT^ and LAGD cell lines were purified and analyzed by SDS-PAGE (Supplementary Figure S3), and the purified enzymes were subjected to N-glycoprofiling of PNGase F released glycans. Site-specific N-glycan analysis after protease digestion by LC-MS/MS was further performed for a subset of the expressed enzymes (Figure 3).

**TABLE 1:** List of lysosomal enzymes included in the study.

Gene	Protein	Disease name	ERT	Molecular form	Monomer molecular weight (kDa)/	N-glycan sites/subunit	Previously published M6P sites	Previously published plasma half-life (mice/rats)	References
GLA	⍺-galactosidase A	Fabry disease	Agalsidase alfa Agalsidase beta	Homodimer	51.2	3	2 sites can carry M6P	12 min (Fabry mice, 1 mg/kg)	Lee et al. (2003), Tian et al. (2019)
AGA	Glycosylasparaginase	Aspartylglucosaminuria (AGU)	na	Heterodimer	24 (α subunit)	1 (α subunit)	Both sites can carry M6P	4 min (AGU mice, 1 mg/kg)	Oinonen et al. (1995), Tikkanen et al. (1995), Dunder et al. (2000)
					17 (β subunit)	1 (β subunit)			
GUSB	β-glucuronidase	Mucopolysaccharidosis (MPS) VII	Vestronidase alfa	Homotetramer	75	4	3 sites can carry M6P	11.7 min (MPS VII mice, 4 mg/kg)	Grubb et al. (2008), Bones et al. (2011), Naz et al. (2013)
CTSD	Cathepsin D	Neuronal ceroid lipofuscinosis	na	Monomer	48	2	1 or both sites can carry M6P	4 h (CLN10 mice, 25 mg/kg)	Sleat et al. (2006), Caval et al. (2019), Marques et al. (2020)
TPP1	Tripeptidyl-peptidase 1	Late infantile neuronal ceroid lipofuscinosis (LINCL)	Cerliponase alfa	Monomer	46	5	2 sites can carry M6P	12 min (LINCL mice, 5 mg/kg)	Wujek et al. (2004), Meng et al. (2012), Caval et al. (2019)
GAA	Acid ⍺-glucosidase	Pompe disease (glycogen storage disease type II	Alglucosidase alfa	Monomer	110	7	2 sites can carry M6P	1.4 h (Sprague Dawley rats, 10 mg/kg)	McVie-Wylie et al. (2008), Khanna et al. (2012)
IDS	Iduronate 2-sulfatase	MPS II, Hunter syndrome	Idursulfase	Monomer	76	8	3.2 mol/mol^a^	2 h (ICR mice, 0.5 mg/kg)	Millat et al. (1997), Garcia et al. (2007), Togawa et al. (2014)

^a^ M6P sites are unknown. M6P contents (mol/mol) were reported previously.

Site-specific N-glycan analysis of AGA, GUSB, CTSD and GAA expressed in CHO^WT^ cells identified major M6P-tagged N-glycans on AGA (Asn38), GUSB (Asn272), CTSD (Asn134 and 263) and GAA (Asn470) (Figure 3A, Supplementary Table S1), however, we were unable to obtain data for two of the glycosites in GUSB (Asn420 and Asn631) and for three in GAA (Asn140, Asn233 and Asn652). Analysis of the respective LAGD-engineered enzymes revealed that the M6P-tagged N-glycans were converted to complex type N-glycans, mainly of biantennary structures with full galactosylation and variable sialylation. Preexisting dominant complex-type N-glycans (AGA Asn308, CTSD Asn134 and GAA Asn882 and Asn925) as well as preexisting high-Man N-glycans (GUSB Asn173 and GAA Asn390) were largely unaltered. Profiling of total released N-glycans from these four enzymes were in agreement with the site-specific analysis (Figure 3B, Supplementary Table S2), and profiling of LAGD-engineered TPP1 and IDS enzymes also indicated loss of M6P-tagged N-glycans and unaltered high-Man N-glycans. Elimination of M6P-tagging also introduced efficient core fucosylation of complex type N-glycans, as expected.

### 3.4 Homogenous glycodesigns enable intact mass analysis of therapeutic glycoproteins

One of the current major limitations for the characterization of intact glycoproteins by native MS is the inherent heterogeneity of N-glycans. The ability to engineer the glycosylation capacities in production host cells makes it possible to produce recombinant glycoproteins with more homogeneous N-glycans, thereby facilitating characterization of the glycoproteome profile by native MS. We previously showed that extensive glycoengineering of erythropoietin resulted in highly homogeneous glycoforms which facilitated the interpretation of the high resolution native MS data (Yang et al., 2015b; Čaval et al., 2018). Here we explored the feasibility of direct native MS analyses of engineered lysosomal enzymes.

**FIGURE 4:**
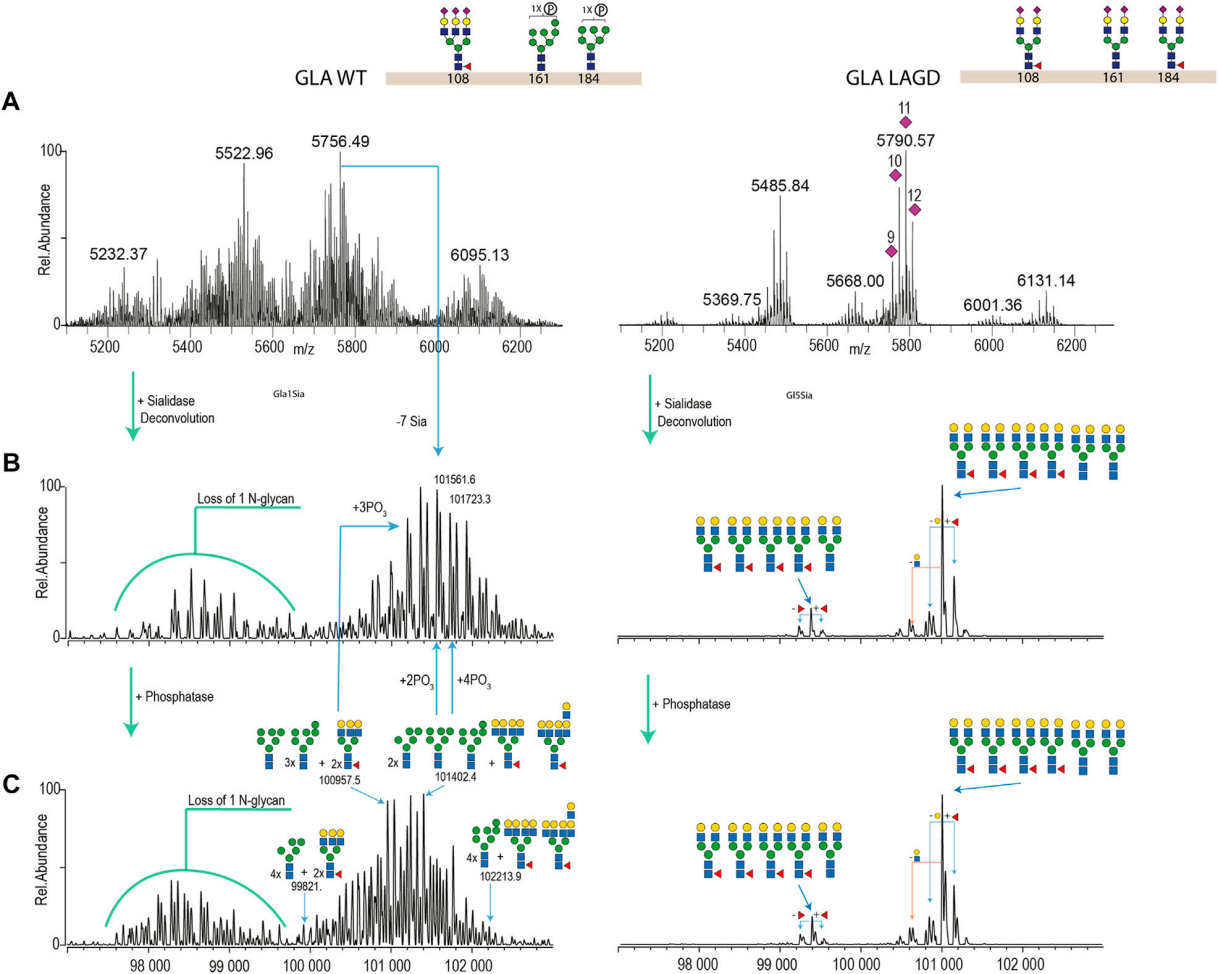
Intact MS analysis of GLA WT/glycodesign at the native conditions and after sialidase and phosphatase treatment. Most abundant glycan structures at N-glycosites (Asn108, Asn161, and Asn184) of GLA WT and GLA LAGD are illustrated next to the enzyme name. Mass deconvoluted spectra of GLA WT and GLA LAGD are shown on the left and right sides, respectively. Panel **(A)** shows native spectra with purple diamonds denoting the total number of sialic acid residues, as validated by sialidase treatment. Panel **(B)** shows deconvoluted spectra of sialidase treated enzyme, while Panel **(C)** shows that of sialidase and phosphatase treated enzyme.

We first analysed GLA expressed in CHO^WT^ cells and in a LAGD-engineered CHO cell line that produces GLA enzyme with homogeneous biantennary N-glycans capped with α2-3 sialic acid (Tian et al., 2019) (Figure 4). Direct analysis of the isolated intact GLA WT resulted in very dense mass spectra, which interfered with the charge deconvolution process necessary to interpret the spectra. Assignment of sialic acid residues and phosphate-moieties could be confirmed by *in vitro* desialylation and dephosphorylation of the protein. Specifically, by determining the glycosylation profile of the desialylated and dephosphorylated GLA WT enzyme (Figure 4C, left) and comparing it against that of the desialylated but phosphorylation-intact GLA WT enzyme (Figure 4B, left), we were able to deduce the phosphorylation pattern. Similarily, by comparing the profiles of the desialylated but phosphorylation-intact GLA WT enzyme (Figure 4B, left) to non-treated GLA WT enzyme (Figure 4A, left), it was possible to obtain the sialylation pattern. In contrast, the GLA LAGD enzyme had a relatively simple profile even in its native state (Figure 4A, right), and analyses following desialylation revealed that the glycosylation is quite homogenous, consisting of four fucosylated biantennary N-glycans and two biantennary N-glycans (Figure 4B, right). No high mannose or phosphomannose glycans were detected in this sample, and biantennary N-glycans were fully capped with α2-3 linked sialic acid, as indicated by the presence of 11–12 Sia per dimer of GLA. A small portion of dimerized GLA is also shown to have only 5-N glycans. In this particular case, native MS was crucial to demonstrate that glycoengineering efforts do not affect dimer formation. Thus, when produced in the LAGD designed host cell, the expressed protein resulted in much less complicated spectra, simplifying the charge deconvolution process and annotations (Figure 4).

**FIGURE 5:**
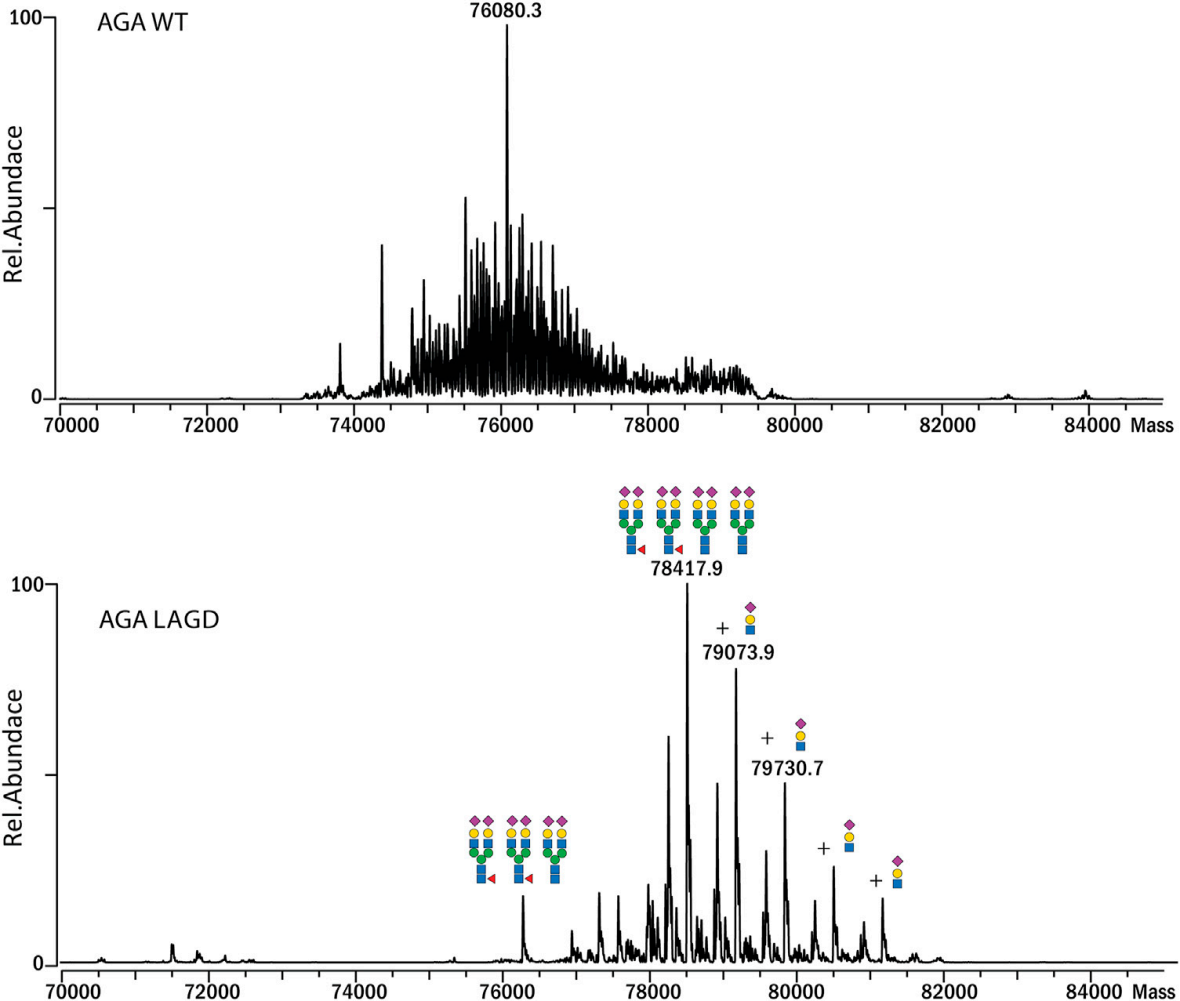
Intact MS analysis of AGA WT/glycodesign at the native conditions. Mass deconvoluted spectra of AGA WT (top) and AGA LAGD (bottom) are shown.

Subsequently, we analyzed MS profiles of AGA expressed in CHO^WT^ cells vs. AGA LAGD and confirmed that the LAGD design enabled straightforward native mass analysis of this enzyme as well (Figure 5). Analyses indicated that AGA LAGD is a heterotetramer of two alpha- and beta-subunits and that the dominant glycoform has fully α2-3 sialic acid capped biantennary N-glycans. Native MS also revealed that both GLA and AGA carry a non-core-fucosylated complex N-glycan. This is in agreement with site specific analysis (Figure 3A) and confirmed that the second N-site of GLA (Asp192) and AGA (Asp285) are non-fucosylated (García-García et al., 2021).

### 3.5 The LAGD glycoengineering design extends circulation time

**FIGURE 6:**
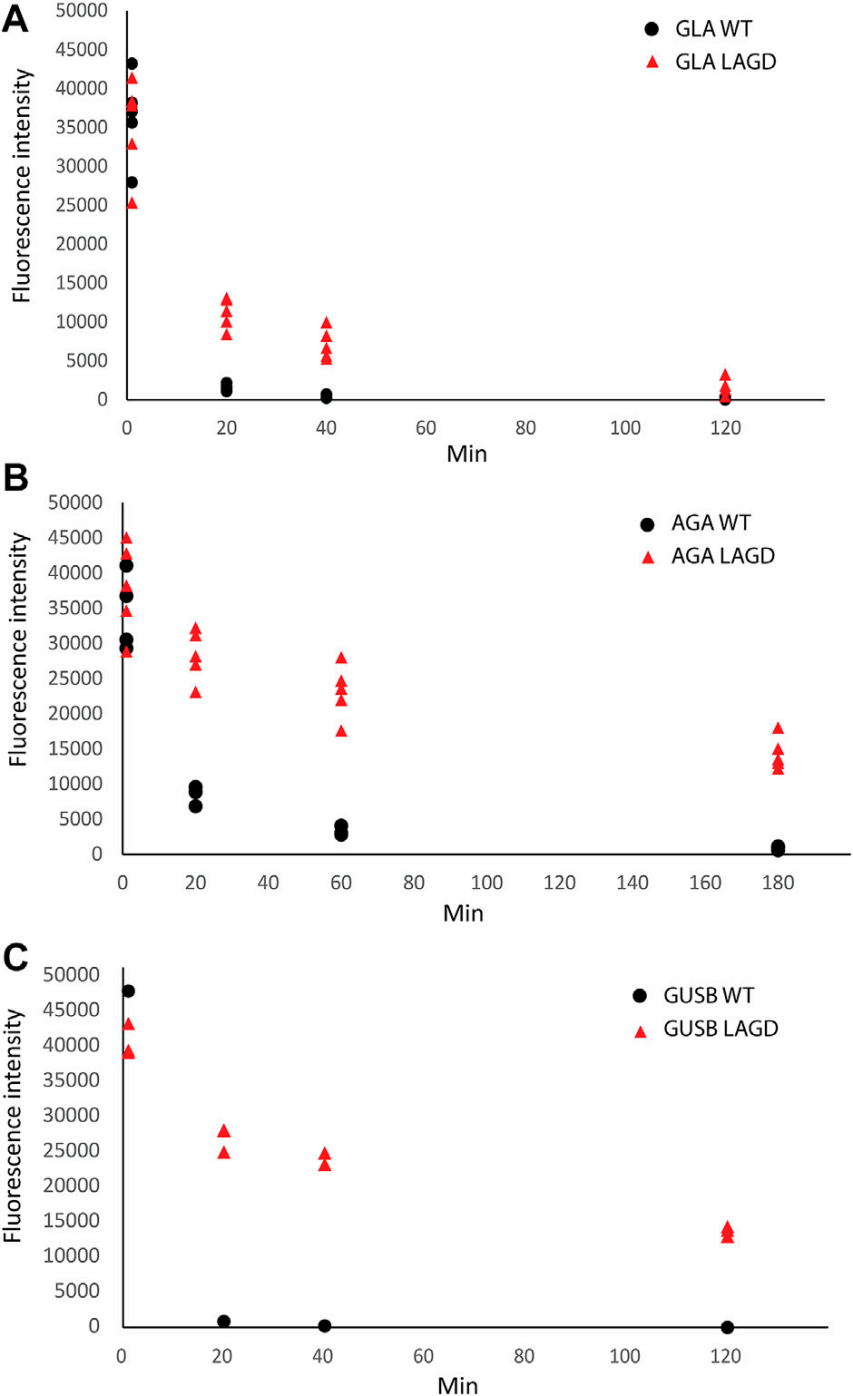
PK study of **(A)** GLA, **(B)** AGA and **(C)** GUSB glycovariants in WT mice. Enzyme activities in plasma, represented as relative fluorescence intensity, after infusion of enzymes are shown. Blood samples were collected at 1, 20, 40, and 120 min after injection of GLA (n = 5 for WT and LAGD) and GUSB (n = 1 and 3 for WT and LAGD, respectively), and at 1, 20, 60, and 180 min after injection of AGA (n = 4 and 5 for WT and LAGD, respectively).

We previously found that elimination of M6P and conversion of N-glycosylation to homogeneous α2-3 sialylated N-glycans increased circulation time of GLA in Fabry mice (Tian et al., 2019). We first tested the performance of GLA WT vs. GLA LAGD and confirmed that the LAGD design improved the circulation time by about three fold (Figure 6A). Interestingly, the calculated half-life was shorter than previously found in Fabry mice, but this may be related to the fact that we used mean GLA activities determined at 1 min in this study compared to 5 min in our previous study as the baseline activity (Tian et al., 2019). We then tested two other enzymes known to have high M6P content, AGA and GUSB. We found the same trends with remarkably enhanced circulation for the LAGD designed enzymes, with an approximately 15-fold extension of half life for AGA and 25-fold extension for GUSB (Figures 6B, C). Note that GUSB has one N-glycan (Asn173) that remains unmodified as high-Man in WT and LAGD enzymes (Figure 3), but this did not appear to affect the improved circulation obtained by the LAGD glycodesign.

## 4 Discussion

Genetic engineering of the glycosylation capacities in mammalian cells offers new opportunities to design therapeutic glycoproteins (Narimatsu et al., 2021). Systematic engineering strategies make it possible to produce a variety of distinct glycoproteoforms of proteins and to study their performance using *in vitro* and *in vivo* assays (Yang et al., 2015b), which may lead to design of new optimal glycoproteoforms for therapeutics. Here, we pursued one of these glycoproteoform designs, LAGD (Tian et al., 2019), for several lysosomal enzymes. We demonstrated that LAGD engineering was broadly applicable to the ERT class of glycoproteins and further showed that the design with α2–3 sialic acid capping provided improved circulation times in mice compared to their wildtype M6P-tagged counterparts.

Engineering glycans of lysosomal replacement enzymes has a long history (Desnick and Schuchman, 2012; Platt et al., 2018), and several lysosomal enzymes in the clinic today are glycoengineered to enhance their therapeutic performance. An example is GBA with homogenous high mannose N-glycans without M6P for targeted macrophage delivery (Mistry et al., 2017). One approach that has yet to reach the clinic aims to entirely eliminate the uptake of replacement enzymes by receptors recognizing the N-glycans inherently found on lysosomal enzymes. Chemical modification of glycans on GUSB (Grubb et al., 2008), SGSH (Gustavsson et al., 2019), TPP1 (Meng et al., 2012) to eliminate glycan receptor interactions has shown promise, and despite loss of M6P and other glycan ligands, these enzymes are taken up by cells and delivered to the lysosome. Moreover, the biodistribution of the enzymes, in particular delivery to the brain, albeit limited, appear to be improved for some of the modified enzymes. In the absence of glycan-mediated receptor uptake, presumably a number of other endocytic receptors, including the low-density lipoprotein receptor (LDLR) related receptors (LRPs) (LRP1 and LRP2) (Markmann et al., 2015; Nielsen et al., 2016), Sortilin (Prabakaran et al., 2012) LIMP-2 (Reczek et al., 2007), and SEZ6L2 (Boonen et al., 2016) substitute and serve in cellular uptake and lysosomal targeting of proteins.

While the LAGD design essentially follows the same concept of eliminating glycan receptor interactions, instead of postproduction chemical treatments that are associated with consistency and compliance challenges, the LAGD strategy involves production of the lysosomal enzymes in mammalian cells with genetically engineered N-glycosylation capacities. The use of stable genetically engineered cells without capacity for tagging glycoproteins with M6P results in production of enzymes with natural N-glycans capped by α2-3 linked sialic acids instead of M6P, and hence these enzymes are designed to avoid binding to and uptake by M6P receptors (Tian et al., 2019). Here, we used repeated infusions of GLA LAGD in Fabry mice to confirm that the engineered enzyme results in prolonged circulation time, and most importantly, in efficient cellular uptake and effective clearance of Gb3 in key organs (Tian et al., 2019). Interestingly, comparable reduction of accumulated Gb3 substrate was achieved between GLA LAGD and Fabrazyme with M6P glycans at the 1 mg/kg dose in all three tissues assessed, while at the lower 0.3 mg/kg dose, GLA LAGD cleared Gb3 to a lesser extent in heart compared to Fabrazyme (Figure 1). This may be interpreted as the GLA LAGD glycoform is less effective. However, since the GLA LAGD enzyme is taken up by cells through a different mechanism than M6PRs, a process that is slower and with substantially lower liver targeting, an alternative interpretation may be that the extended circulation time of the GLA LAGD enzyme provides for wider biodistribution and hence a larger reservoir to deliver and saturate with enzyme. It is predicted that enzymes with N-glycans capped by α2-3 linked sialic acids slowly lose sialic acids with time, likely due to digestion by released lysosomal sialidases (Yang et al., 2015c), which expose the underlying galactose residues and promote uptake and removal by the AMRs in the liver.

Clearance of Gb3 in the kidney was less effective in both Fabrazyme and GLA LAGD infused mice. This may be a result of limited biodistribution of the enzyme to the kidney, but it may also relate to the hydroxylated Gb3 species that are expressed in the kidney and seemingly impervious to ERT. Several studies have explored Gb3 glycolipid profiles in Fabry disease mouse models and the effects of ERTs on these, but the coverage of Gb3 glycolipid species were inconsistent and often not comprehensive (Togawa et al., 2010; Durant et al., 2011; Shin et al., 2018). The most comprehensive study of Gb3 profiles performed on organs from Fabry disease mice found that the di-hydroxylated Gb3 isoforms are quite prevalent in kidney (Ishii et al., 2020). Interestingly, a study previously found that a mono-hydroxylated C24:0 Gb3 isoform was selectively accumulated in the kidney of Fabry mice subjected to ERTs (Kodama et al., 2017). Here, we further found that not only this isoform but all Gb3 isoforms with one and two hydroxylations and varying chain length and unsaturation degrees appeared to be selectively resistant to GLA ERTs (Supplementary Figure S1A). Kidney is the only organ where significant levels of Gb3 is detectable in WT mice (Kodama et al., 2017). We found highly selective reduction in non-hydroxylated Gb3 isoforms compared to both mono- and di-hydroxylated GB3 species in enzyme treated mice (Supplementary Figure S1B). Plausible explanations for this finding may be: i) that the hydroxylated Gb3 species are relatively poor substrates for the GLA enzyme; and/or ii) that the hydroxylated Gb3 species are less accessibly to the GLA enzyme, for example, by expression in different cell types or subcellular localization or by being masked by interactions. In line with the former, the hydroxylated Gb3 isoforms attain conformation changes that are selectively recognized by toxins (Tettamanti et al., 2002).

We previously showed that the LAGD design could be implemented on GLA and GBA (Tian et al., 2019). Here, we showed that the LAGD design can be extended to other lysosomal enzymes (Figure 3). LAGD engineering *via* genetic manipulation converted all M6P-tagged N-glycans to complex sialylated N-glycans, with the notable exception of the preexisting high-Man N-glycans found in GUSB, TPP1, GAA, and IDS. Presumably, such naturally occurring high-Man N-glycans exist because thay are poorly accessible to the enzymes involved in their processing (Xiang et al., 2016). However, despite such persistant high-Man N-glycans, the LAGD design resulted in markedly improved plasma circulatory half-life for the tested lysosomal enzymes (GLA, AGA, GUSB) (Figure 6). This is an interesting finding, as it suggests that the presence of one or more immature N-glycans in glycoproteins otherwise processed normally (e.g., with other fully processed N-glycans) may also not affect interactions with glycan-binding receptors. However, this is likely not the case for glycoproteins with N-glycans that normally are processed to complex type structures and undergo global immature N-glycosylation as may occur in specific cells and diseases (Ugonotti et al., 2021). The present studies further support that lysosomal enzyme uptake mechanisms other than M6PR exist and are efficient.

The blood circulation time of GLA is extremely short due to inherent instability of the enzyme in plasma at neutral pH (Sakuraba et al., 2006; Kizhner et al., 2015), and enzyme variants with greater stability have been developed (Dellas et al., 2021). Clearly, combining the LAGD concept with enzyme engineering would be an interesting pathway to pursue. The LAGD versions of GUSB and AGA are more stable in plasma and showed greatly improved circulation times compared to their respective native proteins (Figure 6). Chemically modified GUSB developed by Grubb et al. (2008) was demonstrated to increase circulation half-life from 11.7 min to 18.5 h in the MPS VII mouse model, while our study in wildtype mice showed increase in estimated circulatory half-life from around 3 min (GUSB^WT^) to 85 min (GUSB^KO^ ^*Gnptab*^). The marked differences in half-life between the two studies may relate to experimental conditions or a genuine difference in clearance. Notably, as discussed above, the GUSB LAGD may lose sialic acids over time and engage in AMR uptake in contrast to the chemically modified GUSB.

Another advantage of glycoengineering is that it provides the opportunity to produce more homogeneous glycoproteoforms that may facilitate production and quality control (Yang et al., 2015b; Narimatsu et al., 2021). Analysis of therapeutic glycoproteins remain challenging with micro- and macro-heterogeneity in glycosylation, often requiring cumbersome bottom-up MS analyses (Denis et al., 1975). The LAGD glycodesigns employed for GLA and AGA provide for limited glycoproteoforms, and therefore, more homogeneity, enabling direct analysis by high-resolution native MS (Figures 4, 5). Finally, it should be noted that lysosomal replacement enzymes can elicit immune reactions, and here we have not addressed potential immunity to the LAGD engineered replacement enzymes.

In summary, our studies demonstrate that LAGD is widely applicable to lysosomal enzymes and may provide opportunities for improved pharmacokinetics, biodistribution and analytics for this important class of therapeutics. While not addressed in the present study, it would be of particular interest to investigate whether the LAGD design can improve delivery of enzymes to the brain in future studies.

## Data Availability

The data presented in the Figure 3, Supplementary Tables S1, S2 are deposited in the proteomeXchange Consortium repository, accession number PXD038783.

## References

[B1] Barton N. W. Brady R. O. Murray G. J. Argoff C. E. Grewal R. P. yu K. (1991). Replacement therapy for inherited enzyme deficiency — Macrophage-targeted glucocerebrosidase for gaucher’s disease. N. Engl. J. Med. 324, 1464–1470. 10.1056/NEJM199105233242104 2023606

[B2] Bern M. Caval T. Kil Y. J. Tang W. Becker C. Carlson E. (2018). Parsimonious charge deconvolution for native mass spectrometry. J. Proteome Res. 17, 1216–1226. 10.1021/acs.jproteome.7b00839 29376659 PMC5838638

[B3] Bones J. Mittermayr S. McLoughlin N. Hilliard M. Wynne K. Johnson G. R. (2011). Identification of N-glycans displaying mannose-6-phosphate and their site of attachment on therapeutic enzymes for lysosomal storage disorder treatment. Anal. Chem. 83, 5344–5352. 10.1021/ac2007784 21599028

[B4] Boonen M. Staudt C. Gilis F. Oorschot V. Klumperman J. Jadot M. (2016). Cathepsin D and its newly identified transport receptor SEZ6L2 can modulate neurite outgrowth. J. Cell. Sci. 129, 557–568. 10.1242/jcs.179374 26698217

[B5] Braulke T. Bonifacino J. S. (2009). Sorting of lysosomal proteins. Biochim. Biophys. Acta - Mol. Cell. Res. 1793, 605–614. 10.1016/j.bbamcr.2008.10.016 19046998

[B6] Brumshtein B. Salinas P. Peterson B. Chan V. Silman I. Sussman J. L. (2010). Characterization of gene-activated human acid-β-glucosidase: Crystal structure, glycan composition, and internalization into macrophages. Glycobiology 20, 24–32. 10.1093/glycob/cwp138 19741058 PMC2782181

[B7] Čaval T. Tian W. Yang Z. Clausen H. Heck A. J. R. (2018). Direct quality control of glycoengineered erythropoietin variants. Nat. Commun. 9, 3342. 10.1038/s41467-018-05536-3 30131559 PMC6104044

[B8] Caval T. Zhu J. Tian W. Remmelzwaal S. Yang Z. Clausen H. (2019). Targeted analysis of lysosomal directed proteins and their sites of mannose-6-phosphate modification. Mol. Cell. Proteomics 18, 16–27. 10.1074/mcp.RA118.000967 30237200 PMC6317476

[B9] Chavez C. A. Bohnsack R. N. Kudo M. Gotschall R. R. Canfield W. M. Dahms N. M. (2007). Domain 5 of the cation-independent mannose 6-phosphate receptor preferentially binds phosphodiesters (mannose 6-phosphate N-acetylglucosamine ester). Biochemistry 46, 12604–12617. 10.1021/bi7011806 17927214

[B10] Couso R. Lang L. Roberts R. M. Kornfeld S. (1986). Phosphorylation of the oligosaccharide of uteroferrin by UDP-GlcNAc:glycoprotein N-acetylglucosamine-1-phosphotransferases from rat liver, Acanthamoeba castellani, and *Dictyostelium discoideum* requires α1,2-linked mannose residues. J. Biol. Chem. 261, 6326–6331. 10.1016/s0021-9258(19)84566-0 2939075

[B11] Dahms N. M. Lobel P. Kornfeld S. (1989). Mannose 6-phosphate receptors and lysosomal enzyme targeting. J. Biol. Chem. 264, 12115–12118. 10.1016/s0021-9258(18)63825-6 2545698

[B12] Dean C. J. Bockmann M. R. Hopwoood J. J. Brooks D. A. Meikle P. J. (2006). Detection of mucopolysaccharidosis type II by measurement of iduronate-2-sulfatase in dried blood spots and plasma samples. Clin. Chem. 52, 643–649. 10.1373/CLINCHEM.2005.061838 16497940

[B13] Dellas N. Liu J. Botham R. C. Huisman G. W. (2021). Adapting protein sequences for optimized therapeutic efficacy. Curr. Opin. Chem. Biol. 64, 38–47. 10.1016/J.CBPA.2021.03.005 33933937

[B14] Denis P. Ossenbergt F. W. Benhamou J.-P. (1975). Hepatic blood flow and enzyme induction in the rat. Biochem. Pharmacol. 24, 249–251. 10.1016/0006-2952(75)90284-1 1111537

[B15] Desnick R. J. Allen K. Y. Desnick S. J. Raman M. K. Bernlohr R. W. Krivit W. (1973). Fabry’s disease: Enzymatic diagnosis of hemizygotes and heterozygotes: α-Galactosidase activities in plasma, serum, urine, and leukocytes. J. Lab. Clin. Med. 81, 157–171. 10.5555/URI:PII:0022214373902837 4683418

[B16] Desnick R. J. Schuchman E. H. (2012). Enzyme replacement therapy for lysosomal diseases: Lessons from 20 Years of experience and remaining challenges. Annu. Rev. Genomics Hum. Genet. 13, 307–335. 10.1146/annurev-genom-090711-163739 22970722

[B17] Dhillon S. (2021). Avalglucosidase alfa: First approval. Drugs 81, 1803–1809. 10.1007/S40265-021-01600-3 34591286

[B18] Dunder U. Kaartinen V. Valtonen P. VäänÄnen E. Kosma V.-M. Heisterkamp N. (2000). Enzyme replacement therapy in a mouse model of aspartylglycosaminuria. FASEB J. 14, 361–367. 10.1096/fasebj.14.2.361 10657992

[B19] Durant B. Forni S. Sweetman L. Brignol N. Meng X. L. Benjamin E. R. (2011). Sex differences of urinary and kidney globotriaosylceramide and lyso-globotriaosylceramide in fabry mice. J. Lipid Res. 52, 1742–1746. 10.1194/JLR.M017178 21747096 PMC3151694

[B20] Flanagan J. J. Rossi B. Tang K. Wu X. Mascioli K. Donaudy F. (2009). The pharmacological chaperone 1-deoxynojirimycin increases the activity and lysosomal trafficking of multiple mutant forms of acid alpha-glucosidase. Hum. Mutat. 30, 1683–1692. 10.1002/HUMU.21121 19862843

[B21] Fraldi A. Biffi A. Lombardi A. Visigalli I. Pepe S. Settembre C. (2007). SUMF1 enhances sulfatase activities *in vivo* in five sulfatase deficiencies. Biochem. J. 403, 305–312. 10.1042/BJ20061783 17206939 PMC1874239

[B22] Garcia A. R. DaCosta J. M. Pan J. Muenzer J. Lamsa J. C. (2007). Preclinical dose ranging studies for enzyme replacement therapy with idursulfase in a knock-out mouse model of MPS II. Mol. Genet. Metab. 91, 183–190. 10.1016/j.ymgme.2007.03.003 17459751

[B23] García-García A. Serna S. Yang Z. Delso I. Taleb V. Hicks T. (2021). FUT8-Directed core fucosylation of N-glycans is regulated by the glycan structure and protein environment. ACS Catal. 11, 9052–9065. 10.1021/ACSCATAL.1C01698/SUPPL_FILE/CS1C01698_SI_001.PDF 35662980 PMC9161449

[B24] Grabowski G. A. Barton N. W. Pastores G. Dambrosia J. M. Banerjee T. K. McKee M. A. (1995). Enzyme therapy in type 1 Gaucher disease: Comparative efficacy of mannose-terminated glucocerebrosidase from natural and recombinant sources. Ann. Intern. Med. 122, 33–39. 10.7326/0003-4819-122-1-199501010-00005 7985893

[B25] Grubb J. H. Vogler C. Levy B. Galvin N. Tan Y. Sly W. S. (2008). Chemically modified β-glucuronidase crosses blood-brain barrier and clears neuronal storage in murine mucopolysaccharidosis VII. Proc. Natl. Acad. Sci. U. S. A. 105, 2616–2621. 10.1073/pnas.0712147105 18268347 PMC2268185

[B26] Grubb J. H. Vogler C. Sly W. S. (2010). New strategies for enzyme replacement therapy for lysosomal storage diseases. Rejuvenation Res. 13, 229–236. 10.1089/rej.2009.0920 20345279 PMC2946059

[B27] Gustavsson S. Ohlin Sjöström E. Tjernberg A. Janson J. Westermark U. Andersson T. (2019). Intravenous delivery of a chemically modified sulfamidase efficiently reduces heparan sulfate storage and brain pathology in mucopolysaccharidosis IIIA mice. Mol. Genet. Metab. Rep. 21, 100510. 10.1016/j.ymgmr.2019.100510 31528541 PMC6737345

[B28] Herzog R. Schwudke D. Schuhmann K. Sampaio J. L. Bornstein S. R. Schroeder M. (2011). A novel informatics concept for high-throughput shotgun lipidomics based on the molecular fragmentation query language. Genome Biol. 12, R8–R25. 10.1186/gb-2011-12-1-r8 21247462 PMC3091306

[B29] Hintze S. Limmer S. Dabrowska-Schlepp P. Berg B. Krieghoff N. Busch A. (2020). Moss-derived human recombinant GAA provides an optimized enzyme uptake in differentiated human muscle cells of pompe disease. Int. J. Mol. Sci. 21, 2642. 10.3390/ijms21072642 32290314 PMC7177967

[B30] Ishii S. Taguchi A. Okino N. Ito M. Maruyama H. (2020). Determination of globotriaosylceramide analogs in the organs of a mouse model of Fabry disease. J. Biol. Chem. 295, 5577–5587. 10.1074/JBC.RA120.012665 32179651 PMC7186183

[B31] Khanna R. Flanagan J. J. Feng J. Soska R. Frascella M. Pellegrino L. J. (2012). The pharmacological chaperone AT2220 increases recombinant human acid a-glucosidase uptake and glycogen reduction in a mouse model of pompe disease. PLoS One 7, e40776. 10.1371/journal.pone.0040776 22815812 PMC3399870

[B32] Kizhner T. Azulay Y. Hainrichson M. Tekoah Y. Arvatz G. Shulman A. (2015). Characterization of a chemically modified plant cell culture expressed human α-Galactosidase-A enzyme for treatment of Fabry disease. Mol. Genet. Metab. 114, 259–267. 10.1016/j.ymgme.2014.08.002 25155442

[B33] Kodama T. Tsukimura T. Kawashima I. Sato A. Sakuraba H. Togawa T. (2017). Differences in cleavage of globotriaosylceramide and its derivatives accumulated in organs of young Fabry mice following enzyme replacement therapy. Mol. Genet. Metab. 120, 116–120. 10.1016/J.YMGME.2016.10.003 27756537

[B34] Koeberl D. D. Luo X. Sun B. Mcvie-Wylie A. Dai J. Li S. (2011). Enhanced efficacy of enzyme replacement therapy in pompe disease through mannose-6-phosphate receptor expression in skeletal muscle. Mol. Genet. Metab. 103, 107–112. 10.1016/j.ymgme.2011.02.006 21397538 PMC3101281

[B35] Lee K. Jin X. Zhang K. Copertino L. Andrews L. Baker-Malcolm J. (2003). A biochemical and pharmacological comparison of enzyme replacement therapies for the glycolipid storage disorder Fabry disease. Glycobiology 13, 305–313. 10.1093/glycob/cwg034 12626384

[B36] Leney A. C. Heck A. J. R. (2017). Native mass spectrometry: What is in the name? J. Am. Soc. Mass Spectrom. 28, 5–13. 10.1007/s13361-016-1545-3 PMC5174146 27909974

[B37] Maresca M. Lin V. G. Guo N. Yang Y. (2013). Obligate ligation-gated recombination (ObLiGaRe): Custom-designed nuclease-mediated targeted integration through nonhomologous end joining. Genome Res. 23, 539–546. 10.1101/gr.145441.112 23152450 PMC3589542

[B38] Markmann S. Thelen M. Cornils K. Schweizer M. Brocke-Ahmadinejad N. Willnow T. (2015). Lrp1/LDL receptor play critical roles in mannose 6-phosphate-independent lysosomal enzyme targeting. Traffic 16, 743–759. 10.1111/TRA.12284 25786328

[B39] Marques A. R. A. Di Spiezio A. Thießen N. Schmidt L. Grötzinger J. Lüllmann-Rauch R. (2020). Enzyme replacement therapy with recombinant pro-CTSD (cathepsin D) corrects defective proteolysis and autophagy in neuronal ceroid lipofuscinosis. Autophagy 16, 811–825. 10.1080/15548627.2019.1637200 31282275 PMC7158922

[B40] Mayes J. S. Scheerer J. B. Sifers R. N. Donaldson M. L. (1981). Differential assay for lysosomal alpha-galactosidases in human tissues and its application to Fabry’s disease. Clin. Chim. Acta 112, 247–251. 10.1016/0009-8981(81)90384-3 6263521

[B41] McVie-Wylie A. J. Lee K. L. Qiu H. Jin X. Do H. Gotschall R. (2008). Biochemical and pharmacological characterization of different recombinant acid α-glucosidase preparations evaluated for the treatment of Pompe disease. Mol. Genet. Metab. 94, 448–455. 10.1016/j.ymgme.2008.04.009 18538603 PMC2774491

[B42] Meng Y. Sohar I. Wang L. Sleat D. E. Lobel P. (2012). Systemic administration of tripeptidyl peptidase i in a mouse model of late infantile neuronal ceroid lipofuscinosis: Effect of glycan modification. PLoS One 7, e40509. 10.1371/journal.pone.0040509 22792360 PMC3391252

[B43] Millat G. Froissart R. Maire I. Bozon D. (1997). Characterization of iduronate sulphatase mutants affecting N-glycosylation sites and the cysteine-84 residue. Biochem. J. 326, 243–247. 10.1042/BJ3260243 9337875 PMC1218661

[B44] Mistry P. K. Lopez G. Schiffmann R. Barton N. W. Weinreb N. J. Sidransky E. (2017). Gaucher disease: Progress and ongoing challenges. Mol. Genet. Metab. 120, 8–21. 10.1016/J.YMGME.2016.11.006 27916601 PMC5425955

[B45] Mononen I. T. Kaartinen V. M. Williams J. C. (1993). A fluorometric assay for glycosylasparaginase activity and detection of aspartylglycosaminuria. Anal. Biochem. 208, 372–374. 10.1006/ABIO.1993.1063 8452235

[B46] Mullis K. G. Kornfeld R. H. (1994). Characterization and immunolocalization of bovine N-acetylglucosamine-1- phosphodiester α-N-acetylglucosaminidase. J. Biol. Chem. 269, 1727–1733. 10.1016/s0021-9258(17)42088-6 8294421

[B47] Narimatsu Y. Büll C. Chen Y. H. Wandall H. H. Yang Z. Clausen H. (2021). Genetic glycoengineering in mammalian cells. J. Biol. Chem. 2021, 100448. 10.1016/J.JBC.2021.100448 PMC8042171 33617880

[B48] Nascimbeni A. C. Fanin M. Masiero E. Angelini C. Sandri M. (2012). Impaired autophagy contributes to muscle atrophy in glycogen storage disease type II patients. Autophagy 8, 1697–1700. 10.4161/auto.21691 22940840 PMC3494606

[B49] Naz H. Islam A. Waheed A. Sly W. S. Ahmad F. Hassan M. I. (2013). Human β-glucuronidase: Structure, function, and application in enzyme replacement therapy. Rejuvenation Res. 16, 352–363. 10.1089/rej.2013.1407 23777470

[B50] Neufeld E. F. (1991). Lysosomal storage diseases. Annu. Rev. Biochem. 60, 257–280. 10.1146/annurev.bi.60.070191.001353 1883197

[B51] Nielsen R. Christensen E. I. Birn H. (2016). Megalin and cubilin in proximal tubule protein reabsorption: From experimental models to human disease. Kidney Int. 89, 58–67. 10.1016/J.KINT.2015.11.007 26759048

[B52] Oinonen C. Tikkanen R. Rouvinen J. Peltonen L. (1995). Three-dimensional structure of human lysosomal aspartylglucosaminidase. Nat. Struct. Biol. 2, 1102–1108. 10.1038/nsb1295-1102 8846222

[B53] Parenti G. Andria G. Ballabio A. (2015). Lysosomal storage diseases: From pathophysiology to therapy. Annu. Rev. Med. 66, 471–486. 10.1146/annurev-med-122313-085916 25587658

[B54] Platt F. M. d’Azzo A. Davidson B. L. Neufeld E. F. Tifft C. J. (2018). Lysosomal storage diseases. Nat. Rev. Dis. Prim. 4, 27. 10.1038/s41572-018-0025-4 30275469

[B55] Poswar F. Vairo F. Burin M. Michelin-Tirelli K. Brusius-Facchin A. Kubaski F. (2019). Lysosomal diseases: Overview on current diagnosis and treatment. Genet. Mol. Biol. 42, 165–177. 10.1590/1678-4685-gmb-2018-0159 31067291 PMC6687355

[B56] Prabakaran T. Nielsen R. Satchell S. C. Mathieson P. W. Feldt-Rasmussen U. Sørensen S. S. (2012). Mannose 6-phosphate receptor and sortilin mediated endocytosis of α-galactosidase A in kidney endothelial cells. PLoS One 7, e39975. 10.1371/JOURNAL.PONE.0039975 22768187 PMC3386966

[B57] Reczek D. Schwake M. Schröder J. Hughes H. Blanz J. Jin X. (2007). LIMP-2 is a receptor for lysosomal mannose-6-phosphate-independent targeting of beta-glucocerebrosidase. Cell. 131, 770–783. 10.1016/J.CELL.2007.10.018 18022370

[B58] Rozaklis T. Beard H. Hassiotis S. Garcia A. R. Tonini M. Luck A. (2011). Impact of high-dose, chemically modified sulfamidase on pathology in a murine model of MPS IIIA. Exp. Neurol. 230, 123–130. 10.1016/j.expneurol.2011.04.004 21515264

[B59] Sakuraba H. Murata-Ohsawa M. Kawashima I. Tajima Y. Kotani M. Ohshima T. (2006). Comparison of the effects of agalsidase alfa and agalsidase beta on cultured human Fabry fibroblasts and Fabry mice. J. Hum. Genet. 51, 180–188. 10.1007/s10038-005-0342-9 16372133

[B60] Sampaio J. L. Gerl M. J. Klose C. Ejsing C. S. Beug H. Simons K. (2011). Membrane lipidome of an epithelial cell line. Proc. Natl. Acad. Sci. U. S. A. 108, 1903–1907. 10.1073/pnas.1019267108 21245337 PMC3033259

[B61] Shaaltiel Y. Bartfeld D. Hashmueli S. Baum G. Brill-Almon E. Galili G. (2007). Production of glucocerebrosidase with terminal mannose glycans for enzyme replacement therapy of Gaucher’s disease using a plant cell system. Plant Biotechnol. J. 5, 579–590. 10.1111/j.1467-7652.2007.00263.x 17524049

[B62] Shen J.-S. Busch A. Day T. S. Meng X.-L. Yu C. I. Dabrowska-Schlepp P. (2016). Mannose receptor-mediated delivery of moss-made α-galactosidase A efficiently corrects enzyme deficiency in Fabry mice. J. Inherit. Metab. Dis. 39, 293–303. 10.1007/s10545-015-9886-9 26310963 PMC4754329

[B63] Shin S.-H. Park M.-H. Byeon J.-J. ill Lee B. Park Y. Ko A. (2018). Chromatography-Quadrupole-Time-of-Flight mass spectrometric assay for the quantification of fabry disease biomarker globotriaosylceramide (GB3) in fabry model mouse. Pharm. A Liq. 10, 69. 10.3390/pharmaceutics10020069 PMC6027126 29880732

[B64] Sleat D. E. Zheng H. Qian M. Lobel P. (2006). Identification of sites of mannose 6-phosphorylation on lysosomal proteins. Mol. Cell. Proteomics 5, 686–701. 10.1074/mcp.M500343-MCP200 16399764

[B65] Stockert R. J. (1995). The asialoglycoprotein receptor: Relationships between structure, function, and expression. Physiol. Rev. 75, 591–609. 10.1152/physrev.1995.75.3.591 7624395

[B66] Surma M. A. Herzog R. Vasilj A. Klose C. Christinat N. Morin-Rivron D. (2015). An automated shotgun lipidomics platform for high throughput, comprehensive, and quantitative analysis of blood plasma intact lipids. Eur. J. Lipid Sci. Technol. 117, 1540–1549. 10.1002/ejlt.201500145 26494980 PMC4606567

[B67] Tamara S. Franc V. Heck A. J. R. (2020). A wealth of genotype-specific proteoforms fine-tunes hemoglobin scavenging by haptoglobin. Proc. Natl. Acad. Sci. U. S. A. 117, 15554–15564. 10.1073/pnas.2002483117 32561649 PMC7355005

[B68] Tekoah Y. Tzaban S. Kizhner T. Hainrichson M. Gantman A. Golembo M. (2013). Glycosylation and functionality of recombinant β-glucocerebrosidase from various production systems. Biosci. Rep. 33, e00071–e00781. 10.1042/BSR20130081 23980545 PMC3782720

[B69] Tettamanti G. Binnington B. Lingwood D. Nutikka A. Lingwood C. A. (2002). Effect of Globotriaosyl Ceramide Fatty Acid-Hydroxylation on the Binding by Verotoxin 1 and Verotoxin 2*, 27. 10.1023/a:1020261125008 12374217

[B70] Tian W. Ye Z. Wang S. Schulz M. A. Van Coillie J. Sun L. (2019). The glycosylation design space for recombinant lysosomal replacement enzymes produced in CHO cells. Nat. Commun. 10, 1785. 10.1038/s41467-019-09809-3 31040271 PMC6491494

[B71] Tikkanen R. Enomaa N. Riikonen A. Ikonen E. Peltonen L. (1995). Intracellular sorting of aspartylglucosaminidase: The role of N-linked oligosaccharides and evidence of man-6-P-independent lysosomal targeting. DNA Cell. Biol. 14, 305–312. 10.1089/dna.1995.14.305 7710687

[B72] Togawa T. Kawashima I. Kodama T. Tsukimura T. Suzuki T. Fukushige T. (2010). Tissue and plasma globotriaosylsphingosine could be a biomarker for assessing enzyme replacement therapy for Fabry disease. Biochem. Biophys. Res. Commun. 399, 716–720. 10.1016/J.BBRC.2010.08.006 20692233

[B73] Togawa T. Takada M. Aizawa Y. Tsukimura T. Chiba Y. Sakuraba H. (2014). Comparative study on mannose 6-phosphate residue contents of recombinant lysosomal enzymes. Mol. Genet. Metab. 111, 369–373. 10.1016/j.ymgme.2013.12.296 24439675

[B74] Ugonotti J. Chatterjee S. Thaysen-Andersen M. (2021). Structural and functional diversity of neutrophil glycosylation in innate immunity and related disorders. Mol. Asp. Med. 79, 100882. 10.1016/J.MAM.2020.100882 32847678

[B75] Vakhrushev S. Y. Dadimov D. Peter-Katalinić J. (2009). Software platform for high-throughput glycomics. Anal. Chem. 81, 3252–3260. 10.1021/ac802408f 19341273

[B76] Wujek P. Kida E. Walus M. Wisniewski K. E. Golabek A. A. (2004). N-glycosylation is crucial for folding, trafficking, and stability of human tripeptidyl-peptidase I. J. Biol. Chem. 279, 12827–12839. 10.1074/jbc.M313173200 14702339

[B77] Xiang Y. Karaveg K. Moremen K. W. (2016). Substrate recognition and catalysis by GH47 α-mannosidases involved in Asn-linked glycan maturation in the mammalian secretory pathway. Proc. Natl. Acad. Sci. U. S. A. 113, E7890–E7899. 10.1073/PNAS.1611213113 27856750 PMC5150396

[B78] Yang Z. Steentoft C. Hauge C. Hansen L. Thomsen A. L. Niola F. (2015). Fast and sensitive detection of indels induced by precise gene targeting. Nucleic Acids Res. 43, e59. 10.1093/NAR/GKV126 25753669 PMC4482057

[B79] Yang Z. Wang S. Halim A. Schulz M. A. Frodin M. Rahman S. H. (2015). Engineered CHO cells for production of diverse, homogeneous glycoproteins. Nat. Biotechnol. 33, 842–844. 10.1038/nbt.3280 26192319

[B80] Yang W. H. Aziz P. V. Heithoff D. M. Mahan M. J. Smith J. W. Marth J. D. (2015). An intrinsic mechanism of secreted protein aging and turnover. Proc. Natl. Acad. Sci. U. S. A. 112, 13657–13662. 10.1073/PNAS.1515464112 26489654 PMC4640737

[B81] Zhou Q. Stefano J. E. Harrahy J. Finn P. Avila L. Kyazike J. (2011). Strategies for neoglycan conjugation to human acid α-glucosidase. Bioconjug. Chem. 22, 741–751. 10.1021/bc1005416 21417264

[B82] Zhu Y. Jiang J. L. Gumlaw N. K. Zhang J. Bercury S. D. Ziegler R. J. (2009). Glycoengineered acid α-glucosidase with improved efficacy at correcting the metabolic aberrations and motor function deficits in a mouse model of pompe disease. Mol. Ther. 17, 954–963. 10.1038/mt.2009.37 19277015 PMC2835178

