# Supplementary material for: A universal GlycoDesign for lysosomal replacement enzymes to improve circulation time and biodistribution
Source: Front Bioeng Biotechnol. 2023 Feb 24;11:1128371. doi: 10.3389/fbioe.2023.1128371 (PMC9999025; doi:10.3389/fbioe.2023.1128371)
Supplement: Supplementary file 2 [file DataSheet1.pdf]

Supplementary material

A Universal GlycoDesign for Lysosomal Replacement Enzymes to Improve Circulation Time and Biodistribution

Yen-Hsi Chen, Weihua Tian, Makiko Yasuda, Zilu Ye, Ming Song, Ulla Mandel, Claus Kristensen, Lorenzo Povolo, André R. A. Marques, Tomislav Čaval, Albert J.R. Heck, Julio Lopes Sampaio, Ludger Johannes, Takahiro Tsukimura, Robert Desnick, Sergey Y. Vakhrushev, Zhang Yang, and Henrik Clausen

Supplementary Figure S1. Summary of Gb3 LC-MS/MS profiling analysis of Fabry mice kidneys.
Supplementary Figure S2. Summary of comprehensive lipidome analysis.
Supplementary Figure S3. SDS Coomassie staining of purified proteins.

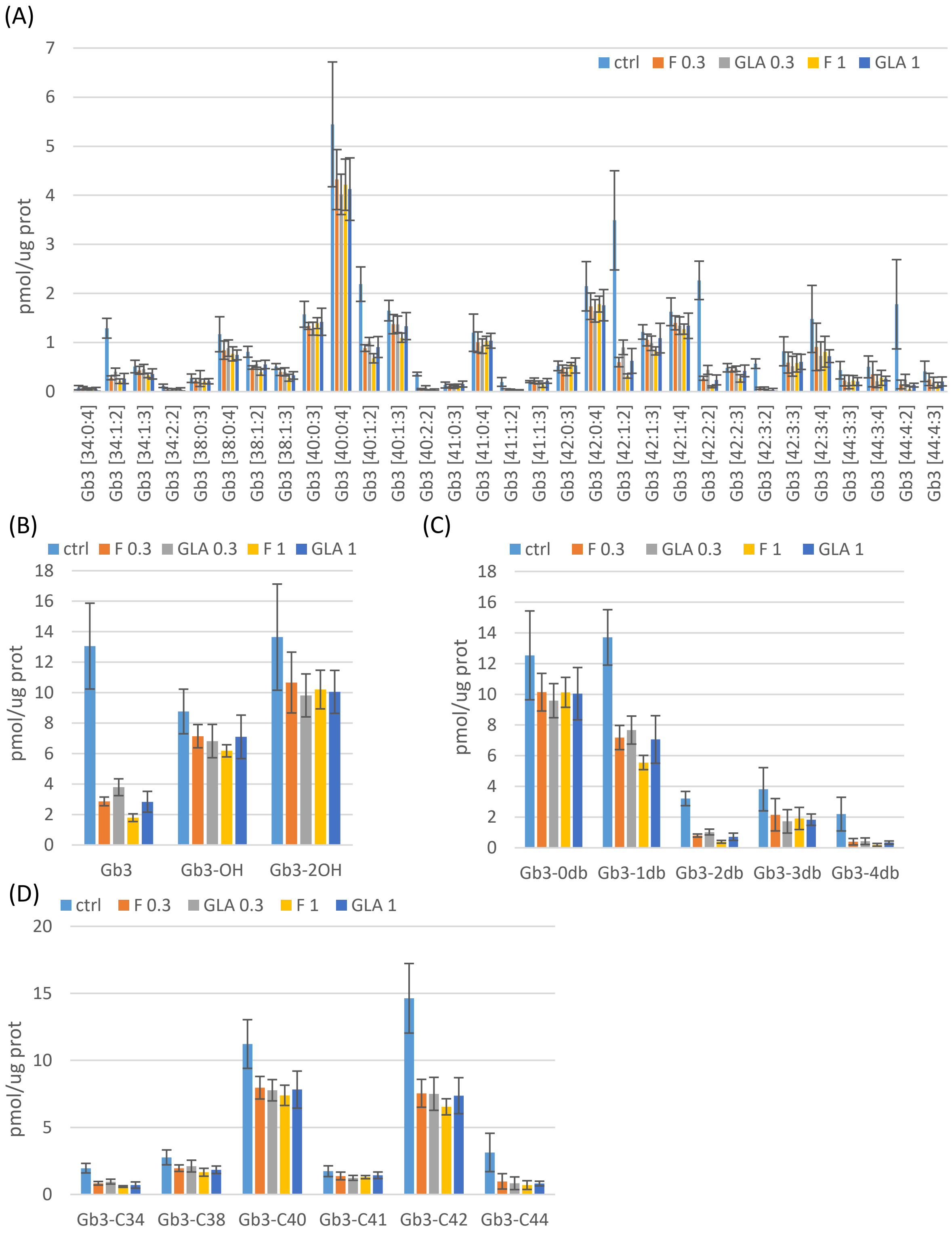

Supplementary Fig. S1. Summary of Gb3 shotgun lipidomics profiling analysis of Fabry mice kidneys. Mice were infused with Fabrazyme 0.3 mg/kg (F 0.3) or 1.0 mg/kg (F 1), GLA LAGD 0.3 mg/kg (GLA 0.3) or 1.0 mg/kg (GLA 1), or saline control (ctrl) every two weeks for 5 times. (A) Quantitation of all Gb3 isoforms present in the kidney. (B) Quantitation of total Gb3 species with and without mono and di-hydroxylation. (C) Quantitation of total Gb3 species with unsaturation lipids. (D) Quantitation of total Gb3 lipid chain length species. Data are the average of five biological replicates and error bars represent the standard deviation of the mean.

A. Kidney lipid class profiles

2-tailed t test

p value	ctrl vs Fabrazyme 0.3	ctrl vs Fabrazyme 1	ctrl vs GLA-Bi23SA 0.3	ctrl vs GLA-Bi23SA 1
DAG	0.118232	0.377036	0.483939	0.911751
PC	0.852513	0.351944	0.288758	0.650586
PCO	0.462719	0.362545	0.581023	0.559253
PE	0.983459	0.226114	0.143312	0.761407
PEO	0.941367	0.344578	0.128247	0.737109
**PG**	0.509378	**0.049955**	**0.024653**	**0.036433**
PA	0.201977	0.811604	0.533127	0.518725
PI	0.811596	0.1491	0.040283	0.282491
PS	0.597687	0.437202	0.030709	0.780823
SM	0.281792	0.10229	0.580922	0.23372
Chol	0.814969	0.517943	0.226208	0.681339
TAG	0.348926	0.063819	0.444802	0.090603
**Cer**	0.131235	0.677953	**0.030252**	0.393129
GlcCer	0.432149	0.799675	0.47897	0.762041
GM3	0.059701	0.17674	0.082428	0.164076
**Gb3**	**3.82E-05**	**8.53E-06**	**9.51E-05**	**0.000153**

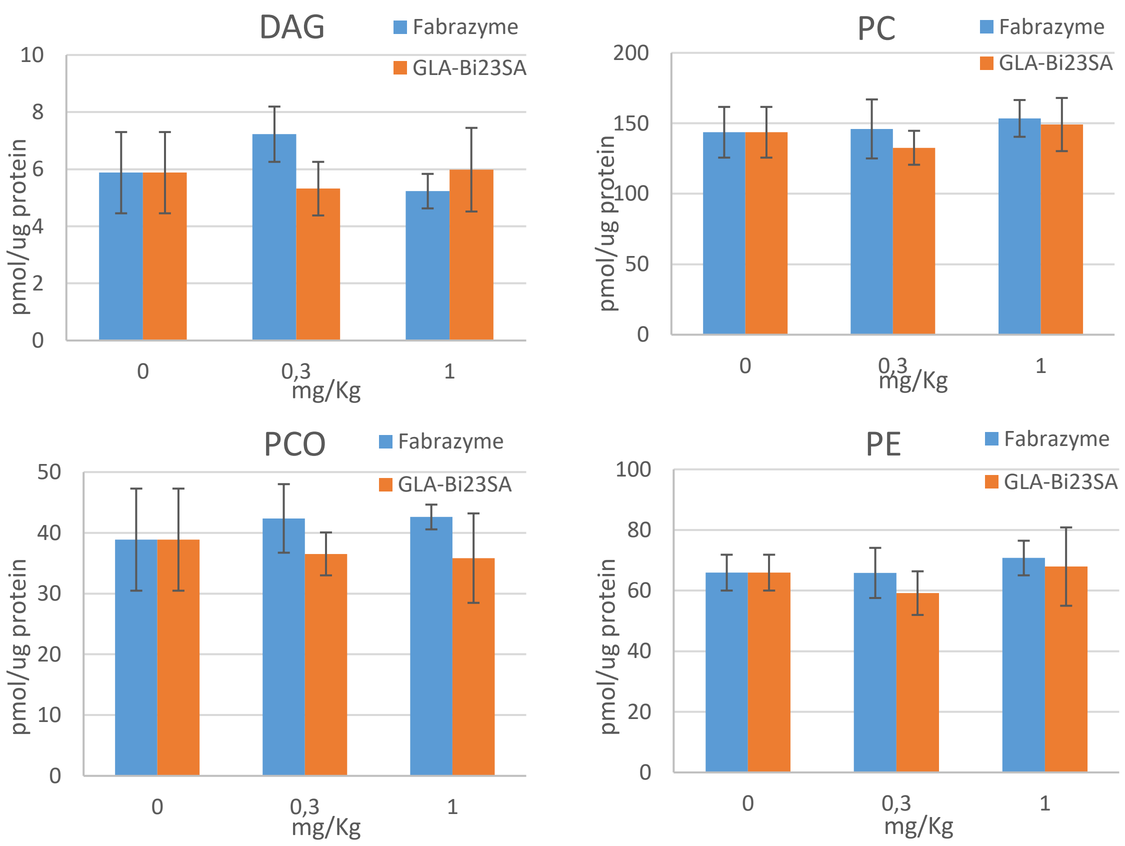

Supplementary Fig. S2. Summary of comprehensive lipidome analysis of organs of Fabry mice. Mice were infused with 0.3 or 1.0 mg/kg Fabrazyme or GLA LAGD (GLA-Bi23SA), or saline control every two weeks for 5 times. (A) Kidney lipid class profiles. (B) Liver lipid class profiles. (C) Heart lipid class profiles. Data are the average of five biological replicates and error bars represent the standard deviation of the mean.

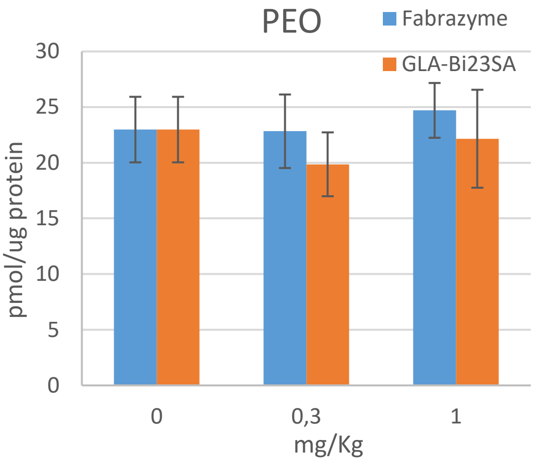
PEO
Fabrazyme
GLA-Bi23SA
pmol/ug protein
0
5
10
15
20
25
30
0
0,3
1
mg/Kg

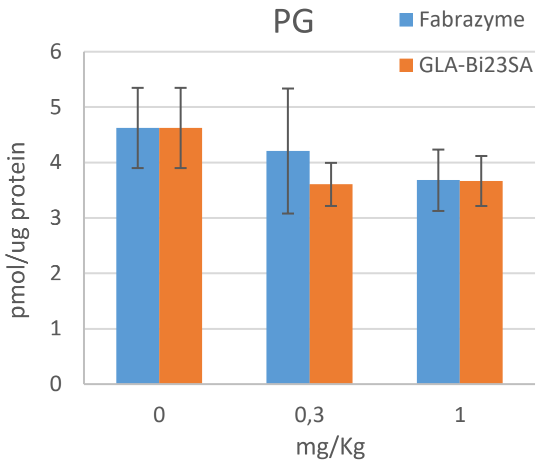
PG
Fabrazyme
GLA-Bi23SA
pmol/ug protein
0
1
2
3
4
5
6
0
0,3
1
mg/Kg

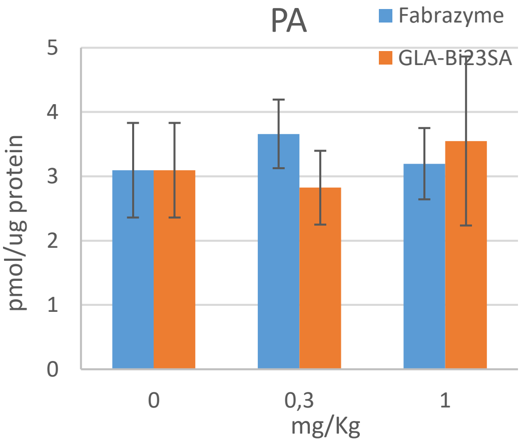
PA
Fabrazyme
GLA-Bi23SA
pmol/ug protein
0
1
2
3
4
5
0
0,3
1
mg/Kg

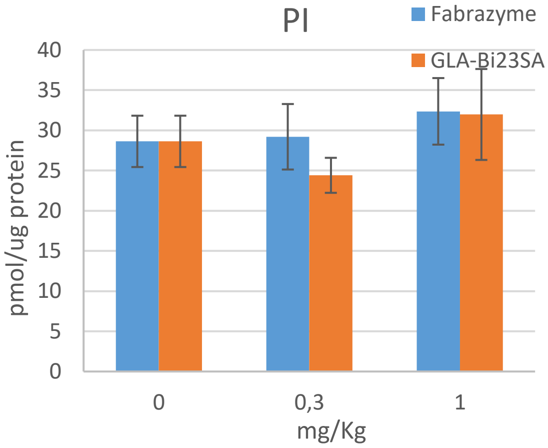
PI
Fabrazyme
GLA-Bi23SA
pmol/ug protein
0
5
10
15
20
25
30
35
40
0
0,3
1
mg/Kg

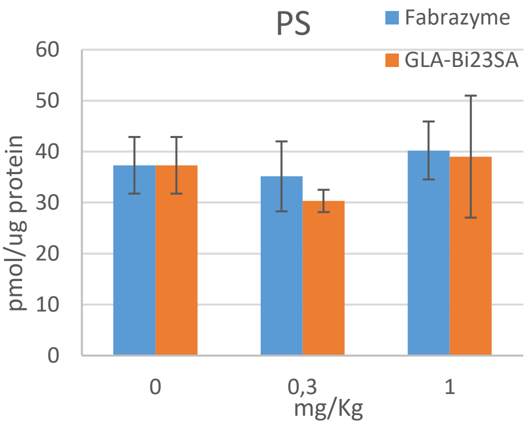
PS
Fabrazyme
GLA-Bi23SA
pmol/ug protein
0
10
20
30
40
50
60
0
0,3
1
mg/Kg

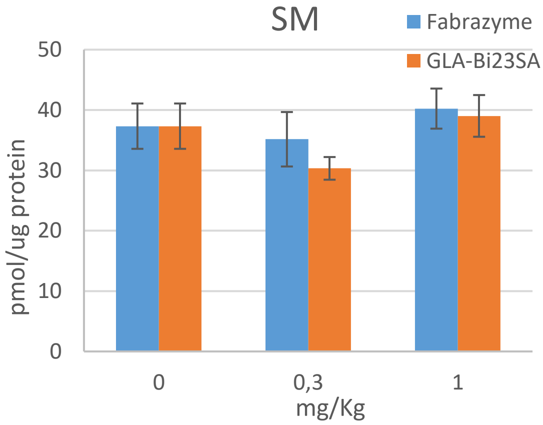
SM
Fabrazyme
GLA-Bi23SA
pmol/ug protein
0
10
20
30
40
50
0
0,3
1
mg/Kg

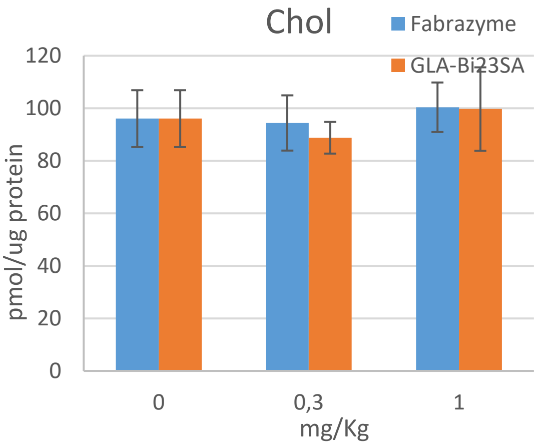
Chol
Fabrazyme
GLA-Bi23SA
pmol/ug protein
0
20
40
60
80
100
120
0
0,3
1
mg/Kg

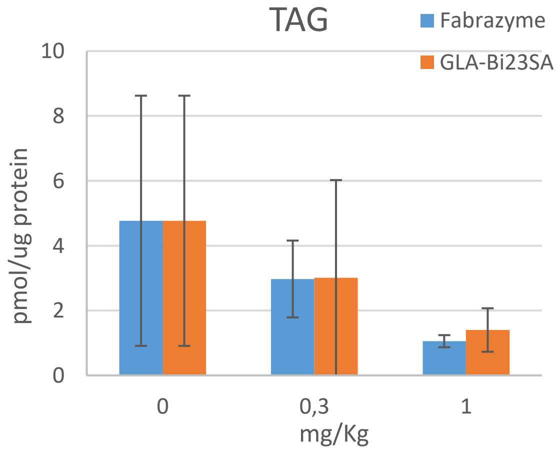
TAG
Fabrazyme
GLA-Bi23SA
pmol/ug protein
0
2
4
6
8
10
0
0,3
1
mg/Kg

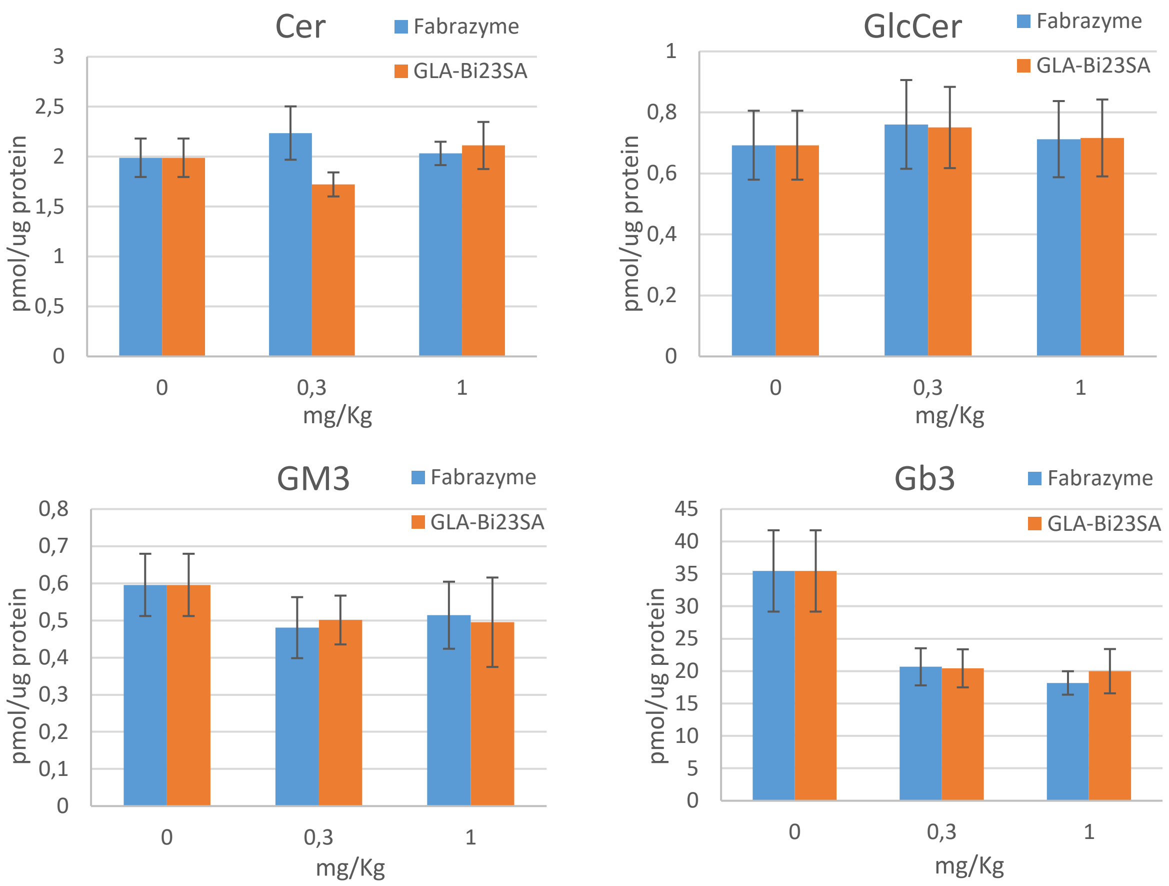

B. Liver lipid class profiles

2-tailed t test

p value	ctrl vs Fabrazyme 0.3	ctrl vs Fabrazyme 1	ctrl vs GLA-Bi23SA 0.3	ctrl vs GLA-Bi23SA 1
DAG	0.149547	0.057328	0.344685	0.056996
**PC**	**0.00786**	0.090175	0.103474	**0.011597**
**PCO**	**0.004177**	**0.009591**	**0.001317**	**0.005631**
**PE**	**0.016931**	0.057091	0.056029	**0.017921**
**PEO**	**0.006094**	**0.021749**	0.195256	**0.03958**
**PG**	**0.000531**	**0.000725**	**0.00135**	**0.000491**
PA	**0.000159**	**0.000584**	0.543259	0.092656
**PI**	**0.005099**	**0.045985**	0.576094	**0.013706**
**PS**	**0.01305**	0.063294	**0.048376**	**0.09517**
**SM**	**0.027947**	0.63207	0.098645	0.091395
**Chol**	**0.003415**	**0.038579**	0.144032	0.077295
CE	0.636914	0.236984	0.31574	0.625119
TAG	0.32759	0.157333	0.518002	0.099665
**Cer**	0.129963	0.066135	**0.046844**	**0.038181**
GlcCer	0.938583	0.61274	0.40784	0.197748
**Gb3**	**0.000472**	**0.00048**	**0.002812**	**1.68E-05**
**lysoGb3**	**Undetected**	**Undetected**	**Undetected**	**Undetected**
GM3	0.15677	0.24295	0.412067	0.275029

Supplementary Fig. S2. continued

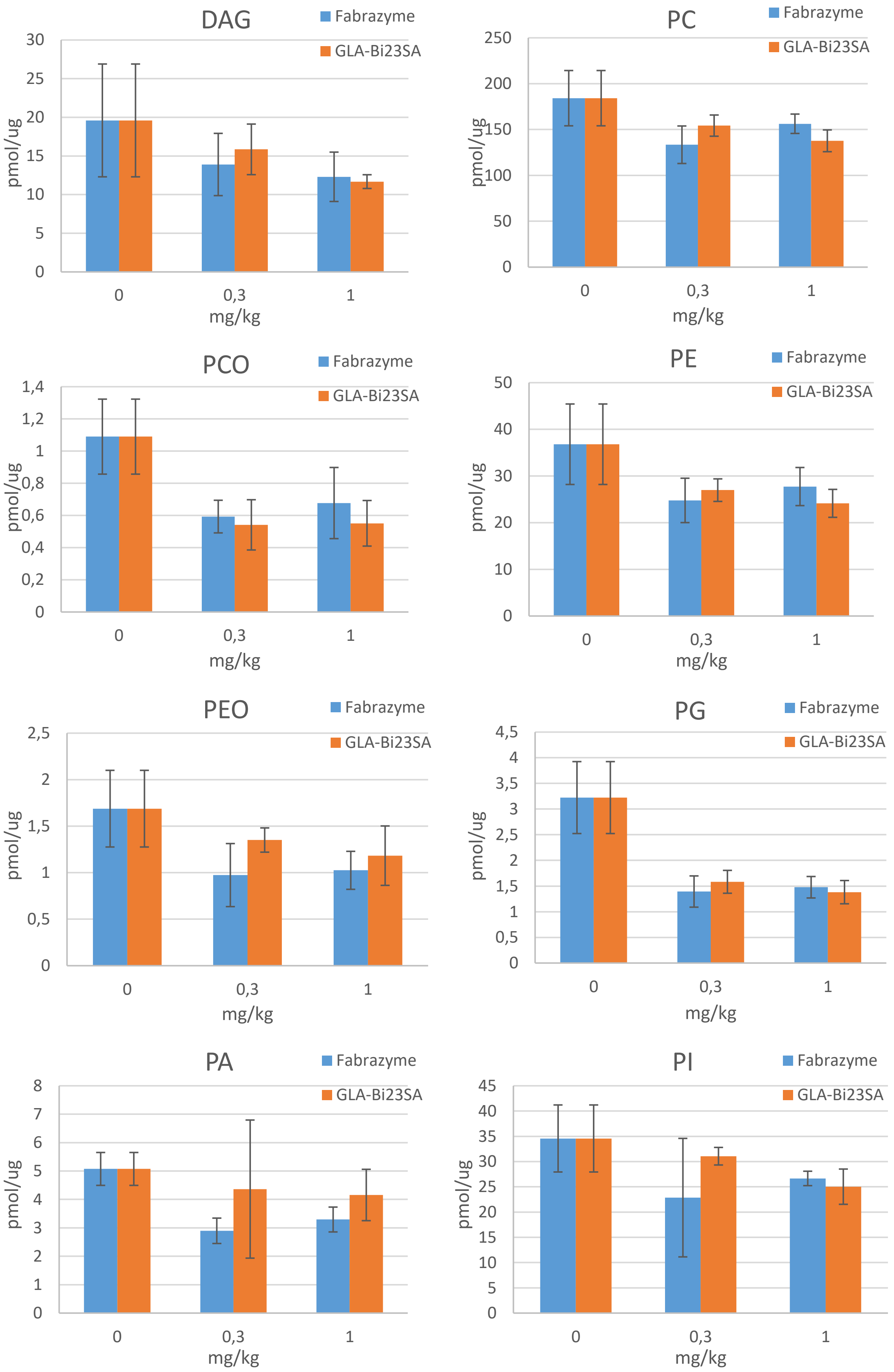

Supplementary Fig. S2. continued

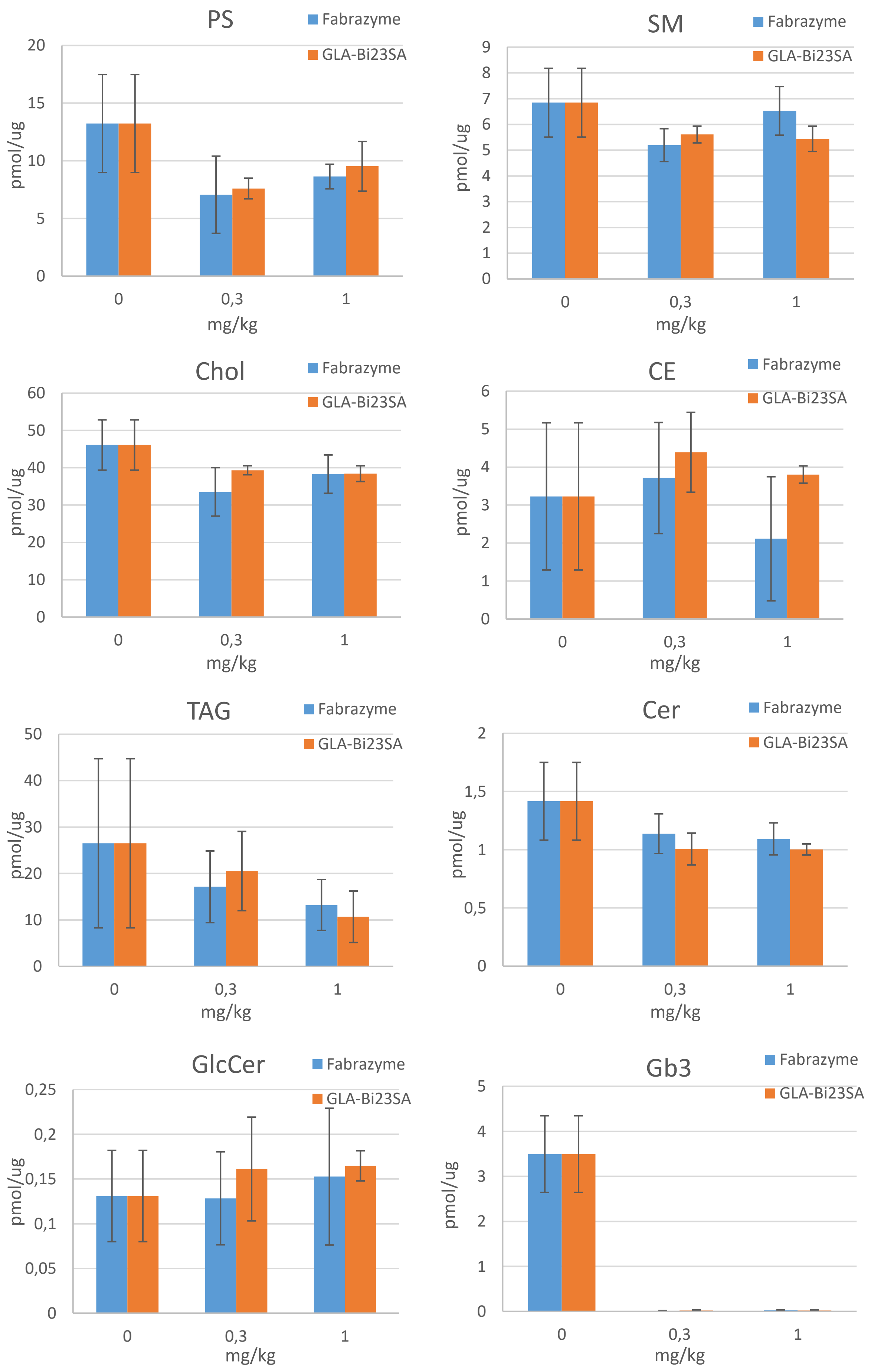
PS
SM
Chol
CE
TAG
Cer
GlcCer
Gb3
Fabrazyme
GLA-Bi23SA
pmol/ug
mg/kg
0
0,3
1

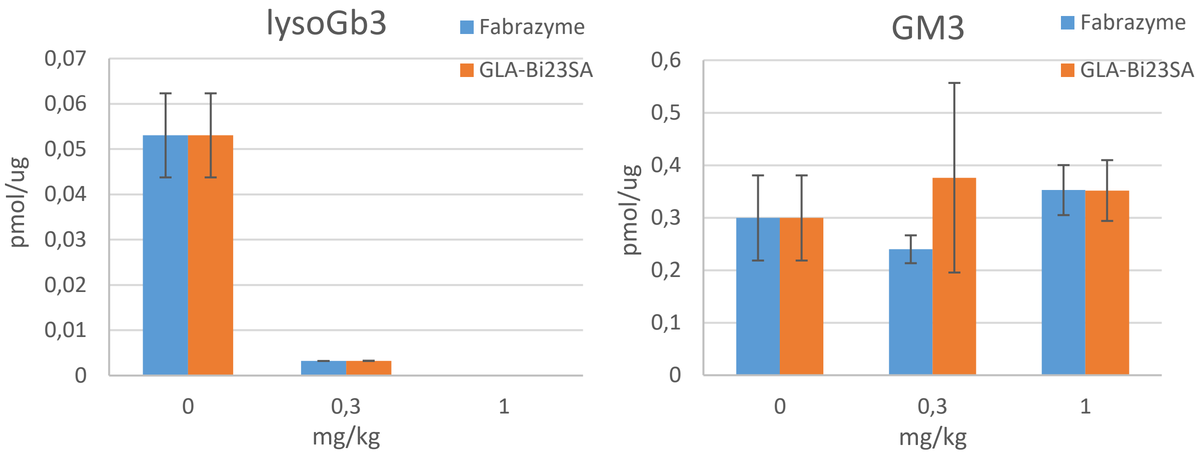

C. Heart lipid class profiles

2-tail test

p value	ctrl vs Fabrazyme 0.3	ctrl vs Fabrazyme 1	ctrl vs GLA-Bi23SA 0.3	ctrl vs GLA-Bi23SA 1
DAG	0.899439	0.936539	**0.02072**	**0.050269**
PC	0.112935	0.611986	0.892941	0.632693
PCO	0.887444	0.718626	0.799871	0.564906
PE	0.058997	0.579922	0.885008	0.969641
PEO	0.610019	0.720463	0.09151	0.348112
PG	0.022878	0.730892	0.616059	0.644764
PI	0.061641	0.410767	0.275419	0.725614
PS	0.083945	0.235295	0.063251	0.160179
SM	0.335075	0.90225	0.765622	0.769891
Chol	0.090155	0.806108	0.401854	0.395056
PA	0.778509	0.172792	0.113249	0.05419
Cer	0.985837	0.208433	0.167756	0.648892
GlcCer	0.266749	0.110881	0.724355	0.01348
Gb3	**1.12E-07**	**1.02E-07**	**4E-06**	**1.14E-07**
GM3	0.203565	0.389229	0.735302	0.18083
CE	0.773058	0.196918	0.53178	**0.006479**
TAG	0.34723	0.089698	0.088214	**0.045936**

Supplementary Fig. S2. continued

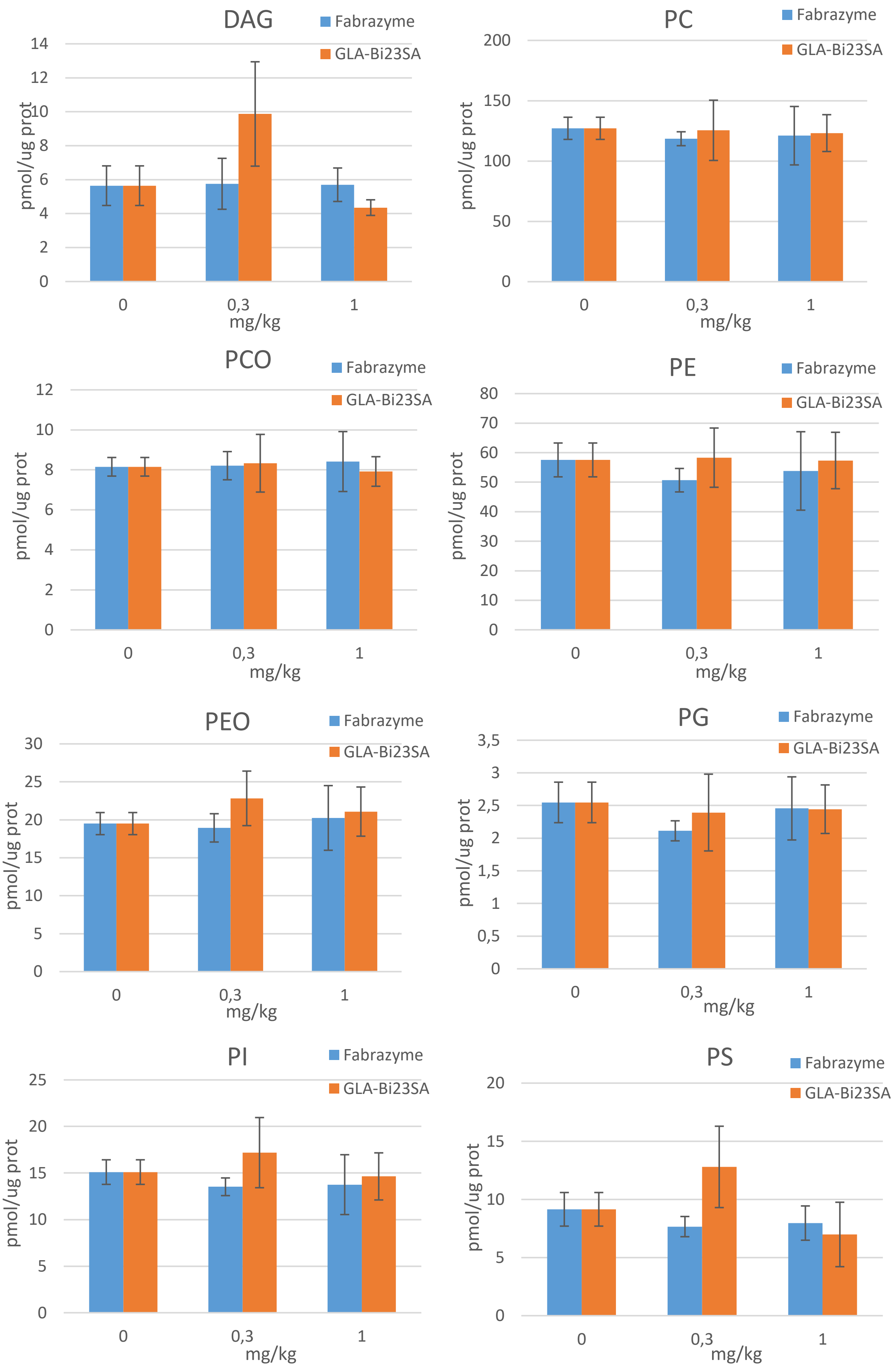

Supplementary Fig. S2. continued

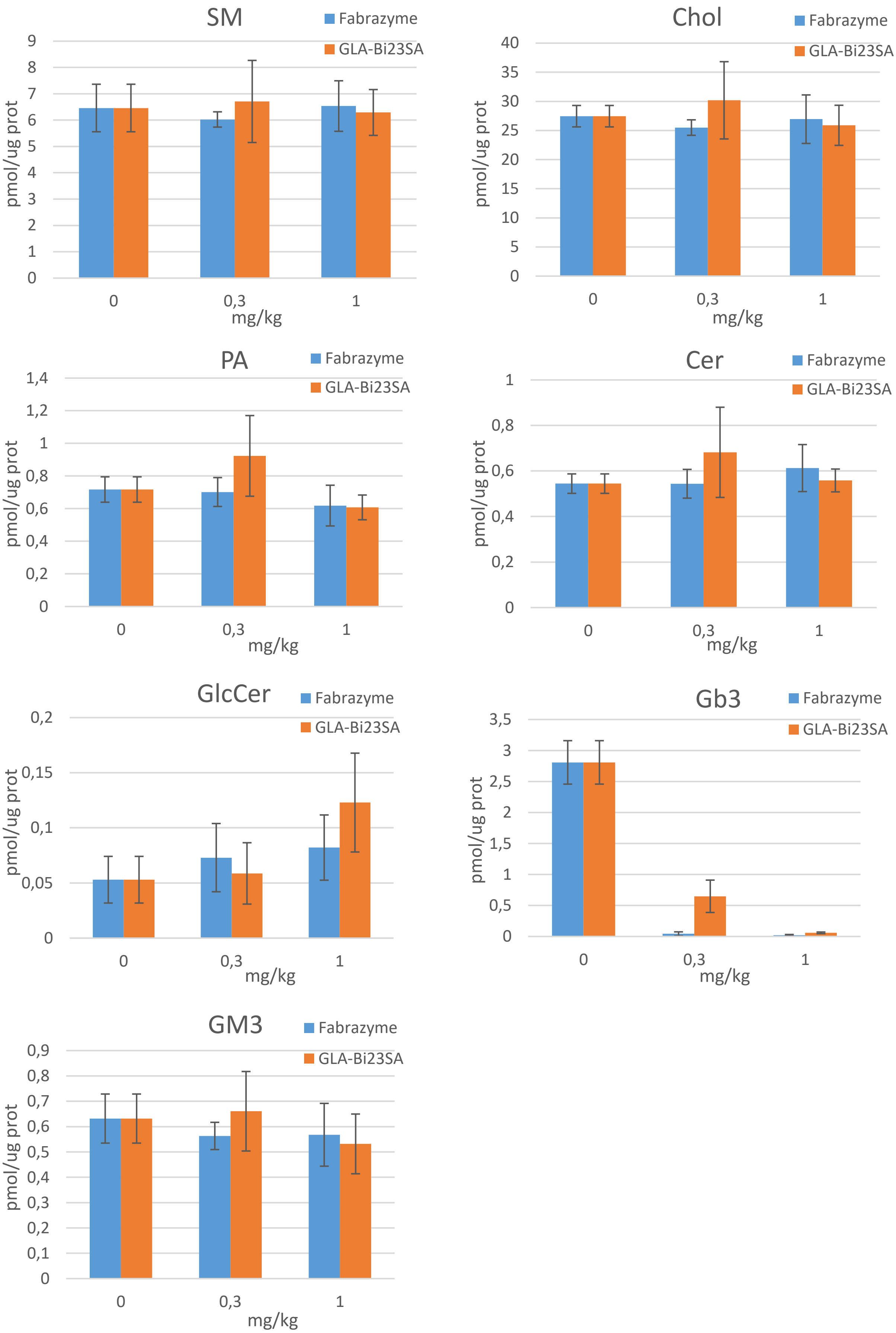

Supplementary Fig. S2. continued

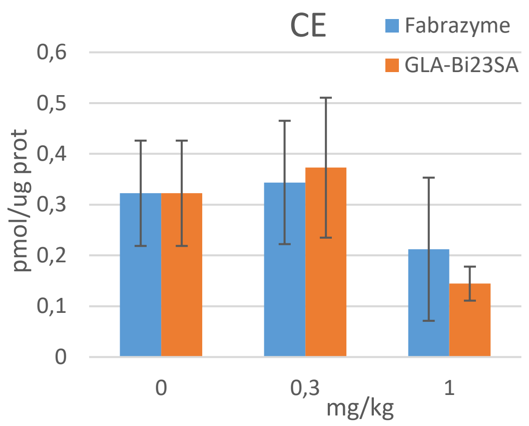
CE
Fabrazyme
GLA-Bi23SA
pmol/ug prot
0,6
0,5
0,4
0,3
0,2
0,1
0
0
0,3
1
mg/kg

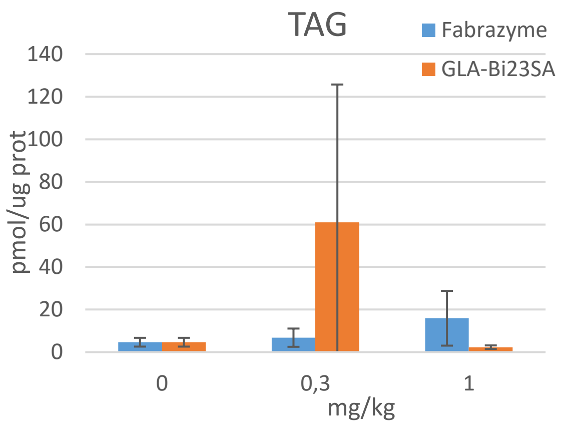
TAG
Fabrazyme
GLA-Bi23SA
pmol/ug prot
140
120
100
80
60
40
20
0
0
0,3
1
mg/kg

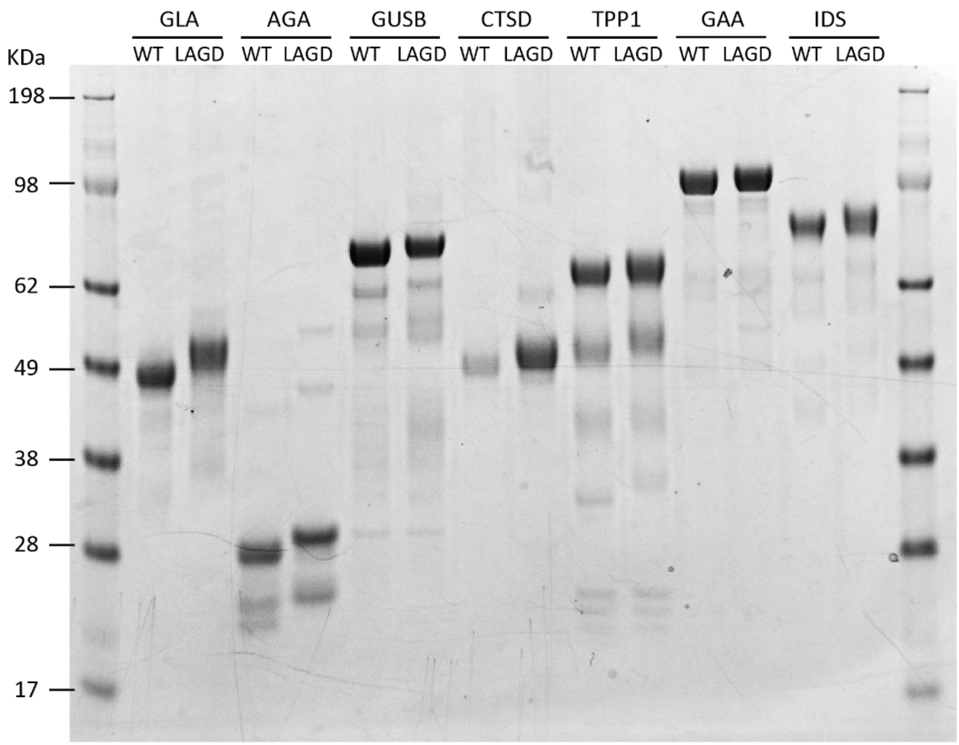

Supplementary Fig. S3. SDS Coomassie staining of purified proteins. Purification yields for enzymes were as follows: AGA WT/ LAGD 25 mg/L; GUSB WT/ LAGD, GAA WT/LAGD, IDS WT/ LAGD and TPPI WT/ LAGD 50 mg/L; and CTSD WT/ LAGD 20 mg/L, based on SDS Coomassie staining.
